# Diachronic reuse of Neolithic burial monuments by Bronze Age newcomers in Southwestern Britain

**DOI:** 10.1038/s41598-026-66094-z

**Published:** 2026-08-26

**Authors:** Nikola Vuković, Carolina Bernhardsson, Hanna Edlund, Gabrielle Delbarre, Martin Smith, Timothy Darvill†, Martin Green, Phillip Endicott, Mattias Jakobsson

**Affiliations:** 1 Human Evolution, Department of Organismal Biology, Uppsala University, Uppsala, Sweden; 2 Department of Archaeology and Anthropology, Bournemouth University, Poole, UK; 3 Down Farm Museum, Dorset, UK; 4 National Museum of Natural History, Paris, France; 5 Institute of Genomics, University of Tartu, Tartu, Estonia; 6 DFG Center for Advanced Studies, University of Tübingen, Tübingen, Germany; 7 Centre for the Human Past and SciLifeLab, Uppsala University, Uppsala, Sweden; 8 Palaeo-Research Institute, University of Johannesburg, Johannesburg, South Africa

**Keywords:** Ecology, Ecology, Evolution, Genetics

## Abstract

The spread of early farming communities first reached Britain c. 4000 BCE, ultimately displacing or assimilating local foragers. Two millennia later, another wave of migration, associated with the Bell Beaker culture, again transformed the genetic and socio-cultural landscape of Britain. Southwestern England is rich in prehistoric burial monuments, some dating from the Early Neolithic, many of which later saw reuse during the Bell Beaker and Bronze Age periods. We analysed the genomes of 30 individuals dated between c. 3800 and 1400 BCE from 12 sites in southwestern England and identified a sharp genetic shift between c. 3000 and 2500 BCE. All individuals predating ~ 3100 BCE were genetically similar to European Neolithic farmers, whilst those after ~ 2550 BCE genetically resembled people associated with the Bell Beaker culture in Britain. At three sites, Bronze Age individuals were buried in monuments originally constructed more than a millennium earlier by genetically distinct Neolithic communities. Lacking clear cultural or biological links to the original builders and users of these monuments, this second wave of burial activity may reflect an attempt by incoming or later groups to anchor themselves within established landscapes and invoke the symbolic authority of earlier burial places. This study provides the first systematic regional ancient genomic transect of southwestern Britain across the Neolithic–Bronze Age transition, an archaeologically rich region previously understudied from a population genomic perspective.

**Supplementary Information:**

The online version contains supplementary material available at 10.1038/s41598-026-66094-z.

## Introduction

Geographically separated from the rest of the continent since c. 8000 years ago^1–3^, the British Isles experienced human migrations and cultural transformations at a different pace compared to mainland Europe. New groups of people brought farming practices across the mainland European continent^4–6^, a migration and transformation that arrived almost a millennium later in the British Isles^7–12^. This transition manifested in the appearance of domesticated crops and animal species^7–9^, pottery^13,14^, and particularly in the construction of large ritual monuments. The earliest and most numerous of these being elongated stone or earthen mounds were constructed during the fourth millennium BCE. These were commonly used as places of collective burial, although their size and prominence in the landscape also denote other significance, most likely as territorial markers^15,16^.

Rather than being a rapid change across the European continent, this was a lengthy and non-uniform process, taking place over thousands of years, moving from the southeast towards the north. For the cultural and genetic changes associated with the British Neolithic to permeate the entire island it required an additional two millennia^17^, resulting in a genetic profile that is a mix between “Neolithic farmer” ancestry (~ 75%) and “Mesolithic forager” ancestry (~ 25%). The transition towards domesticated resources, and later the arrival of metalworking in Britain, both occurred relatively late compared to mainland Europe, likely due to its position at the north-western periphery of Europe.

Around 3000 BCE, nomadic people associated with the ‘Yamnaya’ or ‘Pit Grave’ culture began their migration from the Pontic steppe into Europe^18–22^. Subsequent admixture with local Neolithic groups occurred, often linked with the so-called Corded Ware Culture^19,20^. There followed a new cultural synthesis and expression, termed the Bell Beaker Culture^23^, which reached Britain within a few centuries. In the aftermath of this migration (c. 2400–2000 BCE), Pontic steppe-related ancestry constituted a large fraction of the genetic composition of the people of the British Isles^23^.

The Early Neolithic funerary practice, whereby selected individuals were placed in communal burial structures often referred to as long barrows, went out of use after ~ 3500 BCE and was followed by more than half a millennium during which burials of any kind were relatively infrequent. Following this hiatus there is a transition toward an emphasis on the individual, although, again, a selective approach is apparent, as the numbers of excavated burials can only represent a fraction of the overall population^24,25^. Whilst new forms of round mounds appeared, focused on the burials of single individuals, these were not the only locations for depositing the dead. Repeated examples exist of burials dating from the later third and earlier second millennium BCE, placed as secondary insertions in monuments dating from the Earlier Neolithic that were already over a thousand years old by this time. The continued use of megalithic burial monuments originating in the Neolithic has been observed throughout continental Europe^20,26–32^, but the relationship among the buried individuals through time has been poorly understood.

**Fig. 1:**
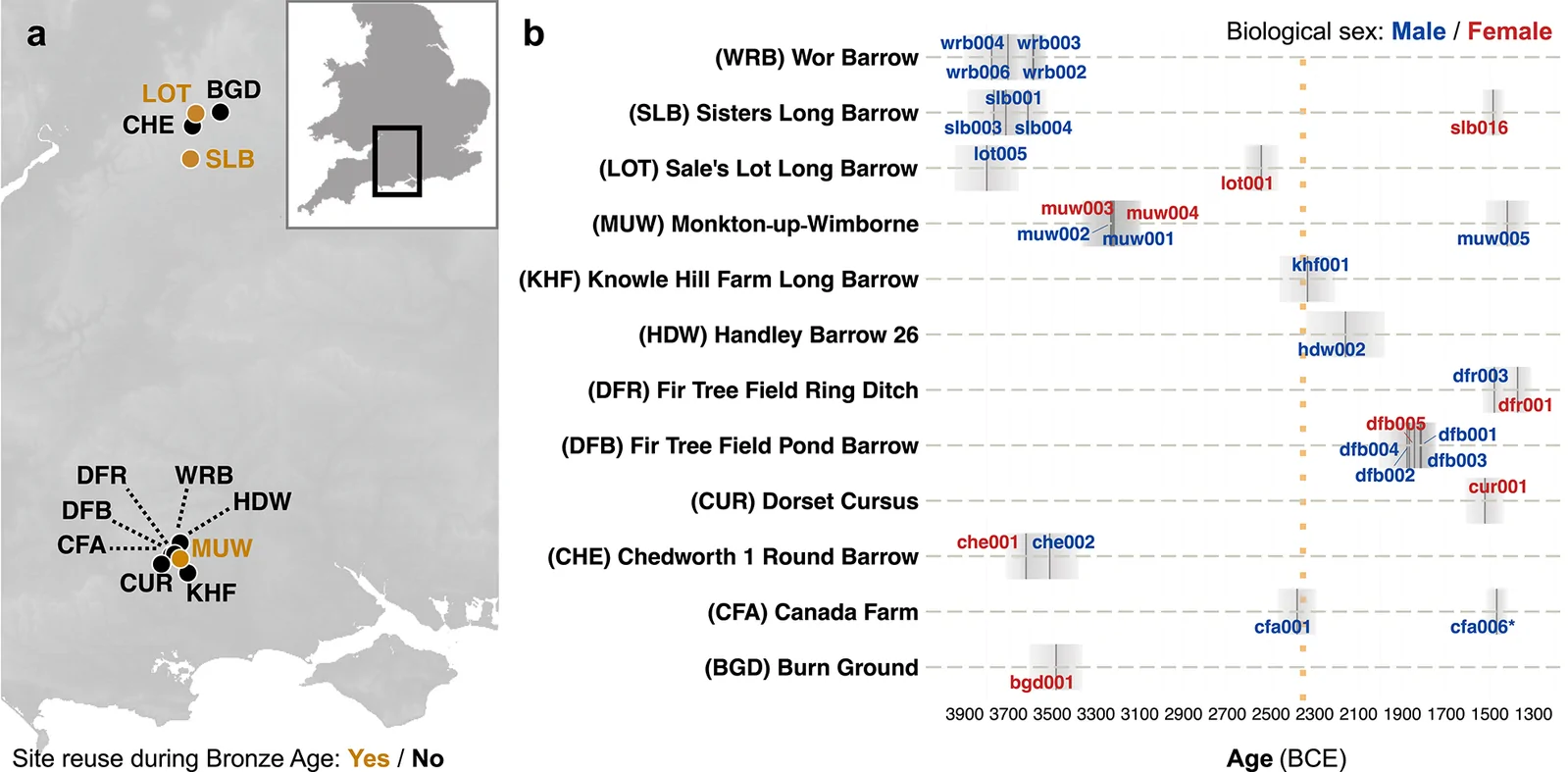
**(a)** Map of southwestern England showing the location of each of the investigated burial sites. Black square on the inset map indicates the location of the study area. (**b)** Radiocarbon-dated individuals in the study per site (y-axis). For each individual, the 95.4% calibrated probability interval is shown in grey. Individual labels on the x-axis are coloured blue (male) and red (female). The orange dotted vertical line represents the point estimate for the Amesbury Archer; its 95.4% calibrated probability interval is 2470–2239 BCE.

To explore population change through time and its relationship to diverse funerary practices in southwestern England, we generated and analysed genomic data from 30 prehistoric individuals from 12 burial sites, spanning c. 3800–1400 BCE. This region is rich in iconic and varied funerary remains, allowing us to trace a major demographic turnover over two millennia in ancient Britain. By combining ancestry estimates with direct radiocarbon dating and site-specific archaeological context, this study directly tests a long-standing archaeological question: whether the secondary reuse of Early Neolithic monuments reflects continuity among local descendant populations or appropriation by newly arrived groups (Fig. 1).

## Material and methods

In total, remains from 30 individuals representing 12 archaeological sites and different cultural contexts were sampled (Table 1). The human remains were curated at the following institutions, which also granted permission to sample the material: Bournemouth University [Burn Ground (bgd001), Chedworth (che001, che002), Sale’s Lot (lot001, lot005), and Sisters Long Barrow (slb001, slb003, slb004, slb016)]; Down Farm Museum [Canada Farm (cfa001, cfa006), Dorset Cursus (cur001), Fir Tree Field Pond Barrow (dfb001, dfb002, dfb003, dfb004, dfb005), Fir Tree Field Ring Ditch (dfr001, dfr003), Handley Barrow (hdw002), Knowle Hill Farm (khf001), and Monkton-up-Wimborne (muw005)]; and Salisbury Museum [Monkton-up-Wimborne (muw001, muw002, muw003, muw004), whose remains were transferred from Down Farm Museum, and Wor Barrow (wrb002, wrb003, wrb004, wrb006)].

**Table 1:** Summary of genetic and archaeological information about the 30 individuals in the study, ordered from oldest to youngest based on radiocarbon dating.

Sample	Archaeological site	Cal BCE (95.4%)	nuDNA coverage	mtDNA coverage	Sex	Mt haplogroup**	Mt cont. (%)	Y haplogroup***
lot005	Sale’s Lot Long Barrow	3800 (3946–3654)	0.13	6.74	XY	K1b1a1	3.72	I2a2a
wrb004	Wor Barrow	3778 (3903–3652)	0.52	50.56	XY	H1	0.71	I
slb003	Sisters Long Barrow	3768 (3888–3647)	0.34	80.79	XY	K1a4a1b	0.41	I2a2a
slb001	Sisters Long Barrow	3713 (3782–3643)	0.11	56.06	XY	J1c3	0.17	I2a2
wrb006	Wor Barrow	3703 (3768–3638)	0.27	9.30	XY	K1b1a1	3.91	I
che001	Chedworth 1 Round Barrow	3621 (3710–3531)	0.28	19.42	XX	W5	1.23	-
slb004	Sisters Long Barrow	3612 (3697–3527)	0.28	76.58	XY	H4a1a1	0.33	I2a2a
wrb003	Wor Barrow	3589 (3647–3530)	0.06	4.84	XY	K1a	4.7	I
wrb002	Wor Barrow	3587 (3645–3528)	0.26	25.28	XY	T2b	0.42	I
che002	Chedworth 1 Round Barrow	3512 (3644–3380)	0.06	3.97	XY	T2	1.58	I2a2
bgd001	Burn Ground	3483 (3603–3363)	0.04	7.00	XX	H	3.72	-
muw002	Monkton-up-Wimborne	3233 (3364–3101)	0.34	37.05	XY	K1a + 195	1.01	I2a2a
muw003	Monkton-up-Wimborne	3232 (3363–3101)	0.11	7.25	XX	K1a	3.33	-
muw001	Monkton-up-Wimborne	3225 (3357–3093)	0.78	107.02	XY	K1a + 195	1.27	I2a2a1a1a2
muw004	Monkton-up-Wimborne	3218 (3340–3096)	0.41	38.07	XX	K1a + 195	0.76	–
lot001	Sale’s Lot Long Barrow	2545 (2622–2467)	0.46	25.44	XX	H6a1b	1.08	-
cfa001	Canada Farm	2381 (2468–2294)	0.24	21.64	XY	HV0 + 195	1.42	R1b1a1a2
khf001*	Knowle Hill Farm Long Barrow	2334 (2461–2206)	0.02	2.07	XY	I1	6.65	R1b1a
hdw002	Handley Barrow 26	2160 (2339–1981)	0.35	49.29	XY	HV + 16,311	0.64	R1b1a1a2
dfb004	Fir Tree Field Pond Barrow	1878 (2008–1748)	0.35	49.52	XY	U5b2a4	0.77	R1b1a1a2a1a2
dfb002	Fir Tree Field Pond Barrow	1868 (1955–1781)	0.66	130.21	XY	I1a1	0.1	R1b1a1a2a1a2
dfb005	Fir Tree Field Pond Barrow	1845 (1920–1769)	0.15	17.85	XX	U5b2a1a2	1.81	–
dfb003	Fir Tree Field Pond Barrow	1818 (1886–1750)	0.36	46.75	XY	T2b2b	0.11	R1b1a1a2
dfb001	Fir Tree Field Pond Barrow	1814 (1882–1745)	0.28	46.38	XY	H1b	0.65	R1b1a1a2
cur001	Dorset Cursus	1523 (1609–1436)	0.04	2.89	XX	T2e	2.62	–
slb016	Sisters Long Barrow	1485 (1534–1436)	0.04	6.20	XX	H1ak1	3.72	–
dfr003	Fir Tree Field Ring Ditch	1480 (1533–1426)	0.35	26.81	XY	H27 + 16,093	1.34	R1b1a1a
cfa006*	Canada Farm	1469 (1516–1421)	0.01	1.80	XY	U5a2b	9.39	R1b1a1a
muw005	Monkton-up-Wimborne	1420 (1519–1321)	0.14	15.11	XY	H	1.96	R1b1a1a2
dfr001	Fir Tree Field Ring Ditch	1373 (1436–1310)	0.21	38.97	XX	U5a2a1	0.25	–

* Used for PCA, excluded from downstream analyses due to low coverage.

** Haplotypes are based on haplogrep v.2.1.16 and positions with a minimum of 3 × coverage. See supplementary data file 3 for full tree and the presence or absence of defining mutations.

*** Haplotypes are based on derived variants where indels, transitions and possible strand-bias positions have been excluded. See supplementary data file 4 for full tree of derived variants from semi-strict criteria (no indels and no transitions).

DNA extraction and library preparation were performed in a dedicated ancient DNA clean room at the Department of Organismal Biology, Uppsala University. DNA extraction was performed on the sampled bone pieces according to the Yang method^33^. Double stranded DNA libraries for Illumina sequencing were prepared from 20 µl DNA extract using the protocol described by^33–35^ with modifications as in^36^. The quality of the libraries was checked using TapeStation 2200 system using High Sensitivity D1000 ScreenTape and reagents (Agilent Technologies) and quantified using Qubit 3.0 Fluorometer (Invitrogen). Uniquely indexed DNA libraries were pooled in equimolar concentrations and whole genome shotgun (WGS) sequenced on either a HiSeq 10X instrument in 150 bp paired-end mode (before August 2019) or a NovaSeq 6000 instrument in 151 bp paired-end mode (after August 2019). All sequencing was performed at the SNP&SEQ facility at NGI-Uppsala, Sweden. Raw fastq files were trimmed and, if overlap between paired end reads, merged using either AdapterRemoval or CutAdapt-Flash. The trimmed and merged fastqs were then aligned to the human reference genome hs37d5 using bwa aln and merged at the library level. Duplicates, reads shorter than 35 bp, and/or reads with < 90% consensus with reference genome, were removed and all per sample library bam files were merged to an individual level. 1240 K transversion positions were called in a pseudohaploidised manner using Samtools mpileup and finally overlapped with the AADR^37^ dataset (v50.0) before downstream analyses. A detailed description of the library preparation, sequencing strategy, bioinformatic processing and downstream population genetic analyses is available in the Supplementary Information file.

## Results

We generated genomic sequence data from 30 ancient individuals (0.26 × average genome coverage) from southwestern England, excavated from different cultural contexts, dated to between 3800 and 1400 BCE (Table 1). The burial types ranged from relatively simple ditches and pits to elaborate long barrows (see Supplementary Information for a more detailed description). The sequenced DNA exhibited signs of post-mortem damage and fragment lengths of < 100 bp, consistent with ancient DNA. A total of 23 individuals had mean nuclear genome coverage greater than 0.1 × . The mitochondrial coverage of all the individuals was > 1 × (median 25.36 ×) and all investigated individuals displayed low levels of mitochondrial contamination (0.10–9.39%). Two individuals (cfa006 and khf001) had low autosomal coverage levels (~ 0.014 and ~ 0.021) and showed the highest levels of mitochondrial contamination (9.39 and 6.65%, respectively). These two individuals were used in the PCA but were excluded from downstream population genomic analyses. Among the 30 individuals, 21 displayed the XY-karyotype (male) and 9 XX-karyotype (female) (Table 1).

**Fig. 2:**
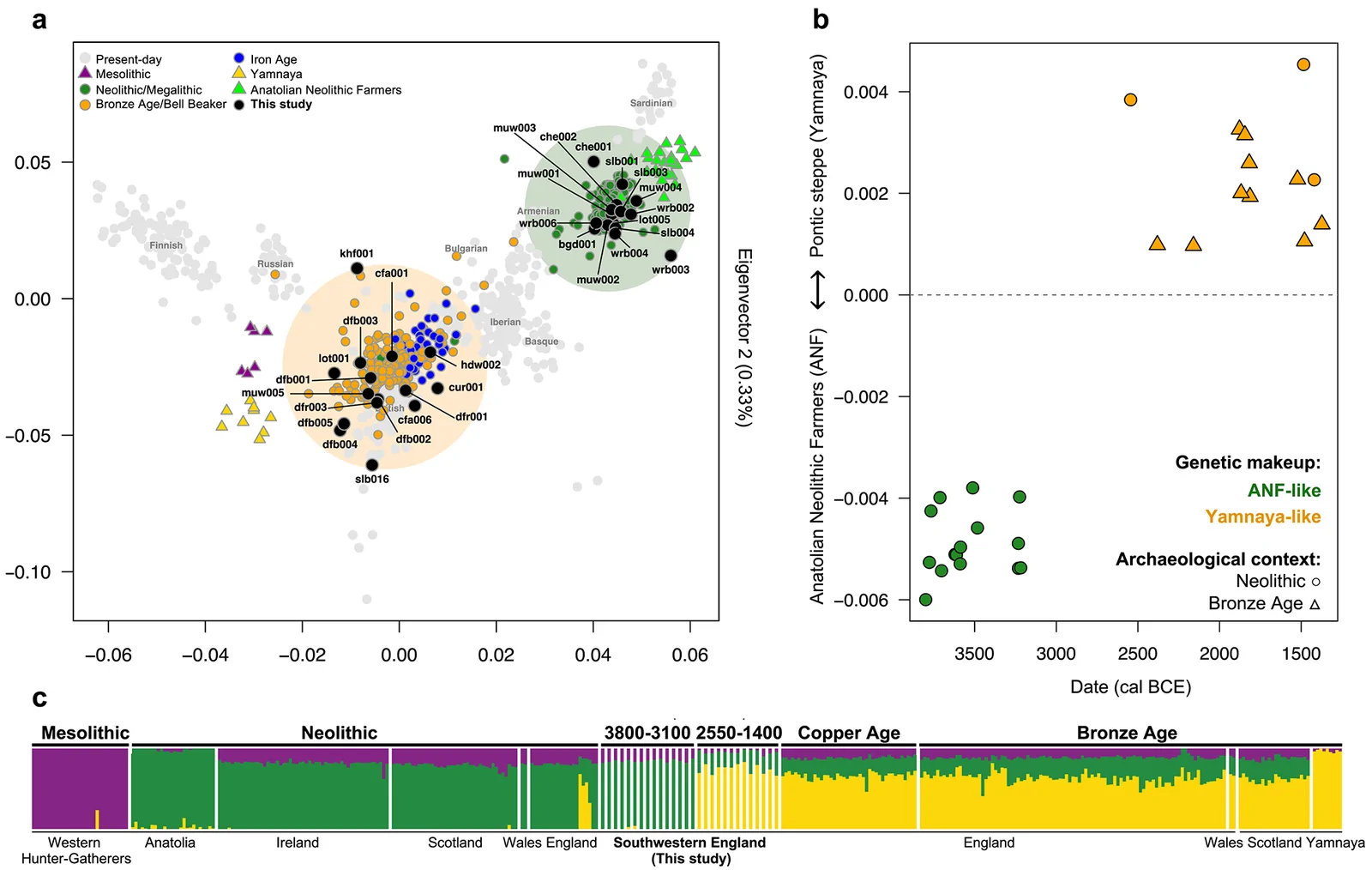
Genetic affinities among the southwestern England individuals. (**Aa)** Principal Component Analysis visualization of the investigated prehistoric individuals from southwestern England and a comparative set of ancient individuals from the British Isles and continental Europe projected on top of a 1240 K panel of present-day Europeans. The individuals are plotted for Eigenvector 1 and 2. Individuals from Neolithic contexts (green circles) form a cluster, and all individuals from southwestern England dated to before 3100 BCE overlap with this cluster. Individuals from Bronze Age and/or Bell Beaker culture contexts form a cluster (orange circles), and all individuals from southwestern England dated more recently than 2550 BCE overlap with this cluster. Two populations, Anatolian Neolithic Farmers (ANF) (light green) and Pontic Steppe Yamnaya-associated individuals (gold) occupy the extremes of each of the two clusters. (**Bb)** Radiocarbon date estimate (x-axis) versus the relative genetic affinity of each test individual to ANF and Pontic steppe Yamnaya-associated individuals (y-axis) (f4(Mbuti, test individual; ANF, Yamnaya)). Colours indicate genetic affinity while symbols reflect archaeological context. (**Cc)** Inferred ancestry components at K = 3, focusing on all ancient individuals from the British Isles, and three proxies of potential source populations (Mesolithic Hunter-Gatherers, Anatolian Neolithic Farmers, and Pontic steppe individuals associated with the Yamnaya culture). Supplementary Fig. 3 shows the full set of investigated individuals and for additional assumptions of the number of ancestry components. Assuming three ancestry components gave consistent results across all 20 replicate runs and the best cross-validation scores (Supplementary Fig. 4).

To visualize genetic similarities and stratification among the individuals from southwestern England and comparative individuals^5,11,17,18,20,22,23,37–52^ from other areas and time periods, we generated a Principal Component Analysis (PCA) plot of the first two PCs (Fig. 2a). The genomic data reveal two distinct clusters among the investigated individuals, broadly overlapping with (i) individuals living in Europe, including Britain and Ireland, during the Neolithic and (ii) individuals living in Europe, including the British Isles, during the Bronze Age, often associated with the Bell Beaker cultural horizon. The southwestern England Neolithic individuals were genetically similar to Anatolian Neolithic Farmers (ANF) and present-day Sardinians. The more recent individuals from southwestern England, dated to the Chalcolithic and Bronze Age, were genetically more similar to other individuals from England dated to the Bronze and Iron Ages as well as present-day Britons. These individuals showed genetic similarity to individuals from the Pontic Steppe, associated with the Yamnaya culture, attributed to large-scale gene flow from the east towards the west during the late Neolithic and early Bronze Age^19,20,23^. These genetic affinities were further quantified by contrasting the affinity of the southwestern England individuals between Anatolian Neolithic Farmers and the Pontic steppe Yamnaya individuals using an f_4_ statistic (Fig. 2b). All individuals dated to before 3100 BCE showed affinity towards ANF (with the majority displaying Z > 3), while all the individuals dated to more recent than ~ 2550 BCE show genetic similarity towards the Pontic steppe Yamnaya individuals (with a majority displaying Z < -3, Fig. 2b).

Genetic ancestry components were estimated using ADMIXTURE^53^ for the southwestern England individuals as well as a large set of comparative individuals from across mainland Europe. The southwestern England individuals that lived between 3800 and 3100 BCE (Table 1) display a large ancestry component (green in Fig. 2Cc) shared with other Neolithic groups and a small ancestry component (purple) shared with Mesolithic Western Hunter-Gatherers (WHGs). The individuals from southwestern England dated to between 2550 and 1400 BCE carry an ancestry component (orange in Fig. 2Cc) also present in other European Bronze Age groups and linked to the migrations from the Pontic steppe (by people associated with the Yamnaya culture), indicating a substantial influx of new people into southwestern England between 3100 and 2550 BCE. The southwestern England individuals between 2550 and 1400 BCE also display modest ancestry components associated with Neolithic Farmers and WHGs. This pattern has been previously observed in other Bronze Age groups, both in the British Isles^23^ and continental Europe^19,20^, indicating gene flow between the newcomers and the locals.

We could model^19,54–56^ the genetic ancestry for British Neolithic individuals coming from two sources (Supplementary Figs. 6–7), with ancestry from an Early European farmer–related group (e.g. LBK_Stuttgart^5^) and Western Hunter-Gatherer-related group (e.g. Loschbour^5^), consistent with the population structure results (Fig. 2). Individuals dated after ~ 2550 BCE require an additional Steppe-related ancestry component (Yamnaya^18,19,22,52^), consistent with new ancestry components arriving with newcomers (Supplementary Figs. 8–9)^23^.

We further tested whether Bronze Age individuals retained ancestry specifically related to local British Neolithic groups by modelling them as a mixture of local Neolithic individuals from this study and continental Bell Beaker-associated individuals from Germany. Most post-2550 BCE individuals were consistent with predominantly Bell Beaker-related ancestry. Individual-level results were compatible with an additional local Neolithic-related component in some cases, suggesting possible local admixture. However, this evidence should be interpreted cautiously, as several estimates were uncertain, with confidence intervals for the local Neolithic-related component overlapping zero and genome coverage varying across individuals (Supplementary Fig. 15).

To examine whether burial communities were structured by biological relatedness, we estimated kinship (READv2^57,58^) among Neolithic and Bronze Age individuals with sufficient SNP overlap. The analysis identified a first-degree kin cluster of four individuals at Monkton-up-Wimborne (muw001, muw002, muw003, muw004; ~ 3360–3100 cal BCE). Two first-degree relationships are confidently resolved as sibling pairs (muw001–muw002 and muw001–muw004; > 40,000 overlapping SNPs) (Supplementary Fig. 1). All four individuals show mitochondrial haplogroup K1a (Table 1 and Supplementary Data), indicating a shared maternal ancestor. The two males (muw001 and muw002) also carry the same Y-chromosome haplogroup (I2a2a, Table 1 and Supplementary Data). Other first-degree pairs could not be classified as sibling or parent–offspring relationships due to low SNP overlap (Supplementary Data File 10).

## Discussion

While genomic analysis of different Neolithic and Bronze Age populations from Britain and Ireland has been conducted before^10,17,18,23,39^, the archaeologically rich area of southwestern England has not been thoroughly examined from a regional perspective. The genomic data from the 30 individuals (from twelve burial sites, covering a temporal transect of c. 2700 years) reveal a pattern of genetic turnover sometime between 3100 and 2550 BCE. This genetic turnover shows a new group of people arriving and bringing a new culture—the Bell Beaker culture—to the British Isles, consistent with previous studies^23^.

### New arrivals: genetic shifts detectable 4500 years ago in southwestern England

The genetic data of the investigated individuals show two distinct genetic affinities separated in time. These two genetic affinities also largely correlate with the osteological classification and archaeological context of the burial sites. However, among the twelve investigated burial sites, three were clearly reused after an extended time period (Monkton-up-Wimborne, Sisters Long Barrow and Sale’s Lot Long Barrow). Having first been used by individuals with a genetic make-up similar to European Neolithic Farmers, later depositions indicate the presence of a different ancestry, genetically similar to that of individuals associated with the Bell Beaker culture. The genetic make-up of the latter group includes ~ 20% ancestry associated with European Neolithic Farmers (Fig. 2Cc). However, since contemporary individuals in mainland Europe display the same pattern, the immigrants could have acquired the genetic ancestry component from Neolithic Farmers prior to arriving in the British Isles. Hence, the observation of a modest Neolithic Farmer-associated genetic component cannot be used to determine local mixing in southwestern England. In accordance with previous studies^10,18,21,23^, we conclude that the dominant ancestry component in all individuals dated to more recent than 2550 BCE traces back to groups living in the Pontic steppe who started expanding into central Europe some five millennia ago^59^.

### Secondary burial at Sale’s Lot Long Barrow, Gloucestershire

The genetic analysis of a female individual (lot001) at the Cotswold-Severn-type Neolithic Long Barrow burial site at Sale’s Lot unveiled a genetic profile matching individuals associated with the Bell Beaker culture (as well as a H6a1b mitochondrial haplogroup)^18,23,60^. The remains were radiocarbon dated to 2622–2467 BCE, resulting in one of the earliest burials in the British Isles of an individual genetically related to the immigrant groups associated with the Bell Beaker culture. Described as “cut into the centre of the core of the barrow” and accompanied by a beaker and a fragment of a copper item, this individual could on one hand represent one of the earliest Beaker burials in Britain. However, an alternative possibility is that the human remains had been curated for an extended period and that at the time of final deposition in the mound, the body was considerably older than the objects placed with it. This latter possibility was evidenced at Canada Farm, Dorset^61^, where a primary burial in a log coffin beneath a round barrow was demonstrated to be considerably older than the associated grave goods, indicating that the respective remains must have been curated for a period spanning generations before burial. Interestingly, the Canada Farm remains produced an almost identical radiocarbon date range to that at Sale’s Lot, with one of two dates obtained being 2620–2470 BCE.

Regardless of which of these possibilities is correct in the case of the Sale’s Lot burial, the individual may well have been a first-generation immigrant to Britain, which might explain the special treatment her remains received in the form of deposition in the ancient long barrow. The fact that this apparently venerated individual was female raises questions regarding both the status of women in early metal-using societies and also the extent to which women actively engaged in migration, rather than males migrating and ‘marrying’ or at least having children with local women. The fact that the Sale’s Lot Beaker burial was inserted into a Neolithic long barrow reveals these incoming groups and their descendants to have been engaging with these monuments from a very early stage after their arrival.

### Family burial at Monkton-up-Wimborne, Dorset, reused 1500 years later

The archaeological description of the Monkton-up-Wimborne middle Neolithic site^62,63^ notes a large, flat-bottomed central feature approximately 10 m wide and 1.5 m deep, enclosed by a ring of 14 widely spaced oval pits forming an arrangement about 35 m in diameter. Excavation of the central feature appears to have been deliberately halted at a natural horizontal joint in the chalk, creating an unusually even floor. This floor was later cut by a tapering shaft about 7 m deep and by an oval grave containing the four burials discussed here. Following deposition, the grave was carefully backfilled with chalk rubble and thoroughly compacted.

Excavated from a single oval grave were the remains of four individuals, contemporaneous with construction of the monument. By studying close genetic relationships among these individuals (muw001, muw002, muw003 and muw004), we found first-degree kinship among two males and two females, including two pairs consistent with full siblings (muw001–muw002 and muw001–muw004).

All four carried mitochondrial haplogroups within K1a, a lineage common in Britain before the genetic turnover associated with the arrival of the Bell Beaker culture^11,18,23^. Based on these observations, together with the overlap in radiocarbon dates (3364–3093 BCE), two relationship configurations are most parsimonious: all four were full siblings, or an adult mother (aged 30–45) and three children (aged approximately 5, 9 and 10 years). Osteological assessment identified one adult female and three subadults. Given that the interments were placed within a constructed feature and subsequently sealed, it remains unclear whether Monkton-up-Wimborne served solely as a family burial site or had broader significance within the social dynamics of Neolithic Britain.

Notably, more than 1500 years after the interment of the Neolithic kin group, the same monument was re-entered for the burial of an unrelated male (muw005; 1519–1321 BCE) whose genetic profile is typical of this period and consistent with Bell Beaker-associated ancestry (Fig. 2). He was placed centrally within the earlier central cut and capped by a small flint cairn, indicating deliberate later reuse. By this stage, the feature may have persisted as a water-holding depression in the landscape, and a rare, broadly contemporary pond barrow was excavated adjacent in the same field^63–65^. Hence, more than 1500 years after the burial of the Neolithic family, a man from a different population and cultural context was interred at Monkton-up-Wimborne.

### Reuse at Sisters Long Barrow, Gloucestershire

A similar pattern of reuse was observed at Sisters Long Barrow. Here the remains of three males (slb001: 3782–3643; slb003: 3888–3647; slb004: 3697–3527 BCE) were deposited amongst a mixed disarticulated assemblage, in a cist constructed within the eastern end of the mound, likely the end result of a complex pattern of burial practice with multiple stages. Two millennia later (1534–1436 BCE) a young adult female (slb016) was interred in a scoop-like grave created in the top of the stone mound. Whilst the earlier depositions displayed genetic ancestry typical of contemporary Neolithic individuals, the latter individual exhibited a genetic makeup similar to other individuals from the period associated with the Bell Beaker culture (Fig. 2).

## Conclusion

While the adaptation and incorporation of ancient tombs into later funerary practices has previously been noted^66^, the extent to which this practice was widespread remains unclear. Here, we identify three cases in which Bronze Age individuals were buried within burial grounds originally constructed during the Neolithic by genetically distinct groups. Comparative data reveal five further sites in the British Isles with individuals directly radiocarbon dated to both the Neolithic (5500–2550 BCE) and the Chalcolithic/Bronze Age (2550–800 BCE), indicating that the later reuse of earlier burial places was not restricted to the sites reported here (Supplementary Fig. 17).

The later return to these burial places, after a substantial gap in funerary use, suggests that already ancient monuments retained social and symbolic significance for later groups, potentially allowing them to anchor themselves within older landscapes or invoke imagined ancestral affiliations. This interpretation should remain cautious, given the limited number of directly dated individuals and burial sites available for comparison.

Our findings may also be affected by sampling and preservation biases. Bell Beaker-associated individuals, with their distinct genetic profile, may be overrepresented if their burials were more archaeologically visible or socially prominent. Conversely, individuals with greater continuity from earlier Neolithic populations may remain genetically underrepresented if their interments were less conspicuous, have not yet been discovered, or were never placed in archaeologically visible burial contexts.

Nevertheless, even if such sampling bias exists, the key observation remains unaffected: the reused Neolithic monuments studied here contain individuals buried more than a millennium later whose ancestry differs substantially from that of the original builders. Current data from other parts of Britain also provide no clear evidence for substantial long-term persistence of groups lacking Steppe-related ancestry^18,21,23^.

More broadly, the reuse of Neolithic megalithic monuments is widely documented across continental Europe^20,26–32^, raising the question of whether similar cases elsewhere reflect local continuity, population replacement, or more complex forms of interaction. The possible role of sex in monument reuse also remains open, as the secondary burials at Sale’s Lot, Sisters Long Barrow and Monkton-up-Wimborne consist of two adult females and one male. However, the sample size is too small to draw firm conclusions.

By integrating genetic and archaeological evidence, we trace a major demographic shift in southwestern England across individual tombs and the wider regional landscape. Although the reuse of early monuments by later communities has long been recognised archaeologically, whether these later individuals were descendants of the original users or members of newly arrived groups has remained debated. Here, we show that this question can be tested directly at both site-specific and regional scales.

In southwestern Britain, profound shifts in ancestry were accompanied by continuity in the use and meaning of place, suggesting that landscapes created by the first farmers acquired a social life beyond their original communities, persisting long after those communities had disappeared.

## Supplementary Information

Below is the link to the electronic supplementary material.

Supplementary Material 1.

Supplementary Material 2.

## Data Availability

The sequence data used in this study is available from European Nucleotide Archive under the accession number PRJEB111359.
