# Supplementary material for: Diachronic reuse of Neolithic burial monuments by Bronze Age newcomers in Southwestern Britain
Source: Sci Rep. 2026 Aug 26;16:26819. doi: 10.1038/s41598-026-66094-z (PMC13518887; doi:10.1038/s41598-026-66094-z)
Supplement: Supplementary file 1 [file 41598_2026_66094_MOESM2_ESM.pdf]

SUPPLEMENTARY MATERIAL

Diachronic Reuse of Neolithic Burial Monuments by Bronze Age Newcomers in Southwestern Britain

Nikola Vuković[1] Carolina Bernhardsson[1] Hanna Edlund[1] Gabrielle Delbarre[2] Martin Smith[2] Timothy Darvill[2] Martin Green[3], Phillip Endicott[1,2,4,5,6] Mattias Jakobsson[1,7,8*]

SITE INFORMATION

Sisters Long Barrow, Baunton, Gloucestershire [SP 03650 05330]

A multi-phase long barrow on the Cotswold Hills the investigation of which started in 2013[1] and is still ongoing. The earliest recognized phase is a passage grave within a rectangular cairn. This was later extended westwards with the addition of a second (lateral) chamber. In the third phase the cairn was extended eastwards and a third (terminal) chamber added to create a Cotswold-Severn type long barrow. The first three phases appear to date to the first half of the fourth millennium BCE. Sometime later, probably in the early third millennium BCE, a fourth constructional phase involved the addition of a cist and low semi-circular platform at the eastern end. Within the cist were the co-mingled remains of 5 or 6 individuals who were alive in the early fourth millennium BCE and thus translocated into this later structure. Secondary burials were added into the eroding mound in the early second millennium BCE as inhumations and cremations.

Chedworth 1 Round Barrow, Chedworth, Gloucestershire [SP 04080 12850]

A single-phase round barrow or 'rotunda grave' on the Cotswold Hills that was fully excavated in 1941[2,3]. The well-made barrow covered a central closed burial cist containing the co-mingled remains of two individuals, both dated to the second quarter of the fourth millennium BCE.

Sale's Lot Long Barrow, Withington, Gloucestershire [SP 04880 15760]

A multi-phase long barrow on the Cotswold Hill that was extensively excavated in 1965[4]. The earliest recognized phase was the remains of a square or rectangular house on the old ground surface towards the eastern end of the site. In the second phase two circular monuments were constructed: a simple passage grave with one open chamber to the east and a round

barrow/'rotunda grave' with a closed central cist to the west[5,6]. Both were probably built during the first half of the fourth millennium BCE, although which came first or whether they were built at the same time cannot be determined. In the third phase the two existing structures were joined together and covered by a roughly trapezoidal cairn to form a Cotswold-Severn style long barrow. An additional lateral chamber was added on the north side of the cairn during this phase which can tentatively be dated to the mid fourth millennium BCE. A secondary burial was cut into the top of the eroding mound in the late third or early second millennia BCE: an inhumation accompanied by an early style low-carinated Beaker and a bronze object of some kind. The excavation records are poor and there is some uncertainty about the origin within the monument of some of the human bones.

Burn Ground, Hampnett, Gloucestershire [SP 10420 16070]

A single-phase long barrow on the Cotswold Hills fully excavated in 1940–1. The trapezoidal barrow in the Cotswold-Severn tradition has an eastern terminal chamber with four side-cells opening from a central passage and, unusually, a cross-passage with two lateral entrances[2,6]. This type of a hybrid form has not been noticed in any other known monuments in the region. Bone was found in nine clusters within the chambers.

Wor Barrow, Sixpenny Handley, Dorset [SU 01200 17300]

A multiphase long barrow on Cranborne Chase fully excavated in 1893–4[7]. The primary phase appears to comprise a timber mortuary house (Konens Høj Type) within a rectangular fenced enclosure[8,9]. This was built between 3685 and 3650 BCE and used for about four generations or approximately 85 years. Two burials (wrb001 and wrb002) were placed first in the mortuary house, lying on their backs. Some years later their legs were folded up to make way for two crouched burials (wrb004 and wrb006). Another burial (wrb003) was then placed near the edge of the mortuary house over burial four, and finally burial five (wrb005) was added. In the second phase, around 3655–3595 BCE the mortuary house was covered by turf and a surrounding ditch was dug in six segments to provide material for a low mound within the Phase 1 fenced enclosure. This was completed by 3635–3485 BCE. In the third phase the ditches were extended to provide material for an enlarged mound thereby creating a Cranborne Chase style long barrow. A man and a child (wrb008 and wrb009) were buried in the ditch at the southeast end. Isotope analysis indicates that everyone had grown up within the region and they had a high animal protein diet.

Handley Barrow 26, Sixpenny Handley, Dorset [SU 01300 17300]

A round barrow on Cranborne Chase that was fully excavated in 1894[7]. The barrow was 12m in diameter and at the time of the excavation stood 0.85m high; it was made of chalk rubble taken from an irregular penannular surrounding quarry-ditch. The remains of two adults were found on the old ground surface under the mound. Two left femurs were carbon 14 dated for this study, providing two dates (SUERC-33328:4410 ±30 BP and BM-2518:3750±50 respectively). Given that DNA was extracted from a petrous bone rather than any of the left femurs, there is an uncertainty which of the dates that are the correct age of the hdw002 individual. However, given the genetic affinity of the individual, clustering with the Bell-Beaker associated individuals in the population genetic analyses, the younger date was chosen as the more probable of the two. At the centre was an unaccompanied inhumation while 2.5m to the west was a male inhumation accompanied by a jet belt-slider. Sherds of Mortlake ware were present in the mound and in the basal fill of the surrounding quarry-ditch. Sherds of collared urn apparently from the body of the mound suggest reuse of the barrow in the early second millennium BCE[10,11].

Canada Farm, Gussage St Michael, Dorset [ST 99270 14488]

A multi-phase round barrow/ring ditch on the chalklands of Cranborne Chase, completely excavated in 2009. Phase 1 comprised the inner ditch, penannular in form with an entrance to the southwest and about 11m in diameter and 0.1m deep. Adjacent to the ditch on the northeast was a 3m square structure formed by nine postholes, the proximity to the burial monument suggests that it might have been a temporary resting place for cadavers prior to burial. A central grave pit (F1) seems to have held a wooden coffin in which was the skeleton of an adult male aged 25–30 years in a semi-flexed position on his left side facing east. Grave goods comprised an S-profile (Wessex Middle Rhine) beaker pot, two pieces of boar's tusk, an antler pendant or toggle, and a flint flake. Dated to 2853-2500 BCE (NZA-34641: 4091±25 BP) and 2468-2294 BCE (SUERC-32210: 3900±30 BP). Phase 2 comprised the outer ditch, 13m in diameter 0.4m deep, probably dug to enlarge the central mound in the mid second millennium BCE. Five further inhumations burials were added: two adolescents (F3 and F4), a child or adolescent (F5), an infant aged 3 or 4 years old (F6), and adolescent or young adult (F8) and a scatter of cremated remains with associated middle Bronze Age pottery. Some of these Phase 2 burials showed evidence that the skeletons had been maintained in anatomical order by drilling tie-holes into selected bones[12,13].

Fir Tree Field Pond Barrow (FTF81), Down Farm, Gussage St Michael, Dorset [SU 00012 14731]

A pond barrow on Cranborne Chase completely excavated in 1981–2[11]. The circular depression ('pond') forming the main element of the barrow was about 20 metres in diameter and scooped into the chalk bedrock to a depth of 0.32m. Earlier pits containing Beaker pottery and associated cultural material had been truncated by the construction of the pond barrow. Activity was widespread across the floor of the barrow including four postholes near the centre associated with the remains of a Food Vessel and radiocarbon dates of 1890-1622 BCE (BM-2327: 3450±50 BP) and 2031-1415 BCE (BM-2192R: 3390±130 BP) and an area of flint-knapping towards the western side. A cluster of burials was found towards the eastern margin. Cremations comprised: F2, remains of an adult and an infant accompanied by a bronze awl and dated to 2337–1645 BCE (BM-2189R: 3620±110 BP); F3, an inverted Food vessel containing the cremated remains of a single adult more than 15 years old dated to 2030–1773 BCE (BM-2326: 3570±40 BP); F10, an inverted plain Food Vessel containing the remains of an adult and an infant; F11, an inverted Collared Urn containing the remains of an infant; F12, an inverted Collared Urn containing the remains of two adults and associated with two bone awls and a bronze awl, dated to 2196–1507 BCE (BM-2190R: 3500±130 BP); F14(C) a collared urn containing the remains of a single adult; and F14 inner fill containing the un-urned cremation of an adult. Inhumations comprised: F6, oval grave with the extended remains of an infant aged 18months to 2 years with a Food vessel over the feet; F14 which contained multiple recuts and (A) fragmentary remains of an infant, (B) fragmentary remains of an infant with unidentified pottery, (D) fragmentary remains of an infant with a Food Vessel. Outside the barrow to the east was a line of three pits, one of which might have held a post.

Fir Tree Field Ring Ditch (DF80), Down Farm, Gussage St Michael, Dorset [SU 00012 14731]

Ploughed out multi-phase round barrow on the chalklands of Cranborne Chase completely excavated in 1980[11]. The Phase 1 comprised a polygonal or segmented ring ditch 15m in diameter that probably enclosed a central barrow. The ditch fill was symmetrical and sealed by two successive stabilization horizons. No burials were associated with phase, although they could have been ploughed out. Flintwork of broadly fourth millennium BCE date was found in the ditch. A lozenge-shaped arrowhead residual in a later context might relate to this phase. Phase 2 involved recutting the original ditch as a continuous ring and adding an outer concentric ditch. No burials were identified with this phase. Peterborough pottery was present in the area and might relate to this phase. Phase 3 comprised a further re-cut of the ditch as a slot whose upper fill included dumps of flint nodules; on the south side, partly overlapping

the edge of the barrow but also extending beyond was a mixed cemetery including cremations and inhumations. A Taunton-phase bronze spiral ring was in the ploughsoil over the southern part of the ring ditch and belongs to this phase in the history of the site. Dated to 1880–1270 BCE (BM-2177R: 3270±120 BP) and 1871–1228 BCE (BM-2178R: 3240±110 BP). Twelve cremations (C1-C12) were identified, seven of them associated with Deverel-Rimbury urns. C6 was dated to 1506–938 BCE (BM-2180R: 3030±110 BP) and C12 was dated to 1421–920 BCE (BM-2179R: 2960±100 BP). Five inhumation burials were recovered, three in a compact group with a posthole that might have acted as a marker. F16 contained an adult male aged 25 to 35. F23 contained the remains of a child aged 3 to 5 years. F105 contained the remains of an adult. F106 contained a juvenile aged 10 to 12. F109 contained an adult female of unknown age. There was a scatter of 11 unburnt human bones over the northern sector of the ring ditch.

Knowle Hill Farm long barrow and Beaker grave, Woodlands, Dorset [SU 03062 10415]

Ploughed out remains of an oval barrow or short long barrow on the chalklands of Cranborne Chase completely excavated in 2016[14]. Two arc-shaped ditch segments were all that remains of the long barrow that is estimated to have been 15–20 long and 10m wide. Southeast of the long barrow a steep-sided and flat-based grave was uncovered (F6), measuring 2.5m by 2m and 0.76 meters in depth. Within the grave traces of a wooden chamber/box were found together with disarticulated human remains of a tall adult male (khf001). Multiple grave goods, including a beaker, stone wrist guard, two arrowheads, fire-lighting kit, and a copper fragment likely to have been part of a knife, were recovered. Dated to 2461–2206 BCE (SUERC-79344: 3862±34).

Monkton-up-Wimborne (MUW97), Down Farm, Monkton-up-Wimborne, Dorset [SU 01310 13670]

Pit grave, shaft and pit circle on the chalklands of Cranborne Chase, fully excavated in 1997[15] (Supplementary Figure 18). Elements of the phasing of this multi-phase site are difficult to resolve but can best be interpreted as a three-stage succession[16,17]. Phase one involved a round depression (rather like a pond barrow) about 10m across and cut 1.5m into the chalk bedrock. Originally it may have been surrounded by a low bank made from the upcast from cutting the depression. An oval grave (F1) was cut into the edge of the depression on the northern edge, partly undercutting the edge of the depression. Four individuals were placed in a crouched position, two at either end: three children and a woman: two girls aged between 5 and 10 years, a boy of about 9 years, and a woman aged about 30. Isotope analysis suggests that the woman grew up on the Mendips some 60km away to the west. Radiocarbon

dates suggest that these people lived in the late fourth millennium BCE: 3514–3101 BCE (OxA-8035: 4585±50 BP). Phase two involved cutting a round shaft c.3.5m across and 7m deep in the southeastern sector of the Phase 1 depression. Upcast from cutting the shaft was used to create a mound or platform on the northwest side of the shaft. Near the base of the shaft was a block of worked and decorated chalk that finds parallels with megalithic art typical of the late fourth and early third millennium BCE in western Britain. Human bone, including fragments of a skull were found in the upper fill of the shaft. Phase three involved a pit circle comprising 14 unevenly spaced pits in a ring (or two adjacent arcs) roughly 35m across that was broadly concentric with the Phase 1 hollow and with a major entrance gap opening to the west and a lesser gap to the east. Finds were few but included a kite-shaped flint arrowhead and fragments of Peterborough Ware.

Dorset Cursus, Gussage St Michael, Dorset [ST 96950 12450 to SU 04050 19200]

A two-phase cursus monument comprising two sections set end to end together extend for a total distance of about 10.4km on a roughly southwest to northeast orientation across the chalklands of Cranborne Chase[11,18,19]. The first phase comprises the western section known as the Gussage Cursus that extends from Thickthorn Down to Bottlebush Down. The second phase's eastern extension is known as the Pentridge Cursus and runs from Bottlebush Down eastwards to Martin Down. Excavations in 1982 and 1984 at Down Farm provide sections at points 700m apart across the Phase 1 monument[20,21]. Four further sections were excavated at the eastern end of the Phase 1 monument in the early 2000s[17]. No excavations have examined the Phase 2 monument. Both cursuses are long narrow enclosures defined by banks and external quarry ditches typically 3m wide and up to 1.5m deep with vertical sides and multiple fills. Their width varies between 90m and 120m. Early radiocarbon dates indicate the presence of residual fifth millennium BCE material in the area 5657–5072 BCE (OxA-628: 6460±140 BP) and 5984–5630 BCE (OxA-627: 6900±100 BP). The construction of the Phase 1 monument in the late fourth millennium BCE is based on three radiocarbon dates of 3366–2936 BCE (BM-2438: 4490±60 BP) from the primary fill, and 3526–3026 BCE (OxA-625: 4575±77 BP) and 3626–2929 BCE (OxA-624: 4570±120 BP) from the secondary fills. Two pieces of human bone were recovered from the secondary ditch fills in layers containing Peterborough Ware during the 1984 excavation; the left tibia and right humerus fragments were considered to come from two separate individuals[21].

Material, DNA extraction and library preparation

Pieces of bone material from teeth, petrous bones, femur and/or vertebra from 30 individuals were sampled at Bournemouth University, Down Farm and Salisbury Museum (Supplementary Data File 1). The analysed individuals were recovered from archaeological sites in Southern England.

Permission to sample the archaeological human remains for this study was obtained from the institutions where the material is curated: Bournemouth University [Burn Ground (bgd001), Chedworth (che001, che002), Sales' Lot (lot001, lot005), and Sister's Long Barrow (slb001, slb003, slb004, slb016)]; Down Farm Museum [Canada Farm (cfa001, cfa006), Dorset Cursus (cur001), Fir Tree Field Pond Barrow (dfb001, dfb002, dfb003, dfb004, dfb005), Fir Tree Field Ring Ditch (dfr001, dfr003), Handley Barrow (hdw002), Knowle Hill Farm (khf001), and Monkton-up-Wimborne (muw005)]; and Salisbury Museum [Monkton-up-Wimborne (muw001, muw002, muw003, muw004; transferred from Down Farm Museum), and Wor Barrow (wrb002, wrb003, wrb004, wrb006)].

DNA extraction and library preparation were performed in a dedicated ancient DNA clean room facility at the department of Organismal Biology, Human Evolution, Uppsala University, containing positive air-pressure and with incoming air passing through HEPA filter. All work spaces are cleaned using sodium hypochlorite (NaOCl) and the lab is regularly UV irradiated (254 nm). In addition, protective clothing was used and all work was performed on safety bench class II.

DNA extraction was performed on the sampled bone pieces according to Yang method[22], with the following modifications: 30-190 mg of bone piece was pre-digested in 1 ml EDTA (0.5M, pH 8) for 30 minutes at 37ºC and thereafter the EDTA solution was discarded[23]. In order to dissolve the bone piece 1 ml lysis buffer (final concentration: 0.45M EDTA, pH 8.0 and 0.2 mg/ml Proteinase K) was added followed by incubation at 37 ºC for 12-18 h. Additional 0.1 mg/ml Proteinase K was added followed by incubation at 55 ºC for 4-5 h. The supernatant was transferred to Amicon Ultra-4 PLTK Ultracel-PL membrane 30kDa filter (Millipore) followed by centrifugation for 10-15 minutes at 5000 rpm. 100 µl was transferred to silica-based membrane for binding and elution of DNA using Minelute purification kit (Qiagen) according to manufacturer's recommendations[22,24,25]. The total volume of each extract was 110 µl (eluted in buffer EB, Qiagen) and extraction blanks were processed as controls.

Double stranded DNA libraries for Illumina sequencing were prepared from 20 µl DNA extract using protocol by[22,24,25] with modifications as in[26]. In addition, the purification steps in the library protocol were performed either using Minelute purification (Qiagen) according to manufacturer's recommendation or AMPure XP beads using a modified protocol retaining shorter fragments (Beckman Coulter). In short, 2.2x of AMPure XP beads was added to the reaction volume after the first blunt-end repair step followed by 5 min incubation. Thereafter 265 µl of 85% EtOH was added to the bead-sample solution and mixed followed by magnetic separation of the beads. This was followed by EtOH wash and elution according to manufacturer's recommendations. Total elution volume was 20 µl. The purification after the adapter ligation was performed according to the manufacturer's recommendation using 1.8x of beads to the reaction volume. In order to determine the number of cycles for library amplification, qPCR was performed (CFX Connect 96 BioRad). A total volume of 25 µl, containing 1 µl of DNA library, 1X Maxima SYBR Green qPCR mastermix and 200 nM each of primer IS7 and IS8[27] were set up in duplicates. Amplification was performed in duplicates in a total of 50 µl per reaction using either single or dual indexing. The duplicates were pooled and purified using AMPure XP according to manufacturer's recommendations (Beckman Coulter). The quality of the libraries was checked using Tapestation 2200 system using High Sensitivity D1000 screentape and reagents (Agilent Technologies) and quantified using Qubit 3.0 Flourometer (Invitrogen). Uniquely indexed DNA libraries were pooled in equimolar concentrations and whole genome shotgun (WGS) sequenced on either HiSeq 10X machine in 150bp paired-end mode (before August 2019) or on NovaSeq 6000 machine in 151bp paired-end mode (after August 2019). All sequencing took place at the SNP&SEQ facility at NGI-Uppsala, Sweden.

Sequence analysis

For trimming and merging overlapping paired-end reads from the raw FastQ files, we used either AdapterRemoval v. 2.1.7[28,29] (if sequenced before August 2019) or CutAdapt (v. 2.3,)[30] – Flash v.1.2.11[31], (if sequenced after August 2019). The non-default parameters used for AdapterRemoval were --qualitymax 60 --gzip --trimns --collapse. In order to mimic the Adapter Removal settings as close as possible, the non-default parameters used for CutAdapt-Flash were set as follows - Cutadapt: --nextseq-trim 15 --trim-n -e 0.2 --minimum-length 15:15. FLASH: --min-overlap 11 --max-overlap 150 --allow-outies --compress-prog=gzip --quiet. In both cases, all reads which passed per library and unique sequence run were merged into a single fastq file and mapped against hs37d5 with BWA aln[32] and parameter settings -l 16500 -n 0.01 -o 2. Finally, the bam files were merged to library level and a modified version of FilterUniqSAMCons_cc.py) was used to collapse duplicated reads (with the same start and

end positions). Reads shorter than 35 base pairs (bp), with a mismatch rate > 10% to the reference genome or a lower mapping quality than 30 were discarded. By merging the 90percent consensus library bam files together, a single bam file was created for each individual.

Ancient DNA authentication

To authenticate the DNA of the ancient individuals, the distribution of read lengths was assessed by randomly extracting the read lengths of 2 million reads with a mapping quality > 30 using Samtools view (version 1.5)[32,33]. Additionally, the terminal deamination patterns of the fragments were determined by calculating the fraction of C -> T substitutions at the 5´end and G-> A substitutions at the 3`end of the fragments (See Supplementary Data File 2).

Sex determination, uniparental haplogroups and contamination estimates

Sample contamination estimates were obtained using two different mitochondrial DNA based methods[34,35] where an individual's Mitochondria reads are compared to 311 mitochondrial reference sequences (Supplementary Data File 2). Biological sex was determined using the ratio between sequences that align to the X compared to those aligning to the Y chromosome[36].

Samtools mpileup v1.5 (parameters set at -q30 -Q30) and vcfutilis.pl vcf2fq (parameters set at -d 3) was used to generate strict mitochondrial sequences[32,33] and HaploGrep[37] v. 2.1.16 was used to assign mitochondrial haplogroups, which employs 5,437 mitochondrial haplogroups from Phylotree 17[38] (See Supplementary Data File 3).

For Y-haplogroup estimation, Y chromosomal positions overlapping ISOGG (v.11, April 2016) were extracted using Samtools mpileup v1.3 (parameters set at -B -q30 -Q30)[33]. Derived variants resistant to strand misidentification (no A/T or G/C mutations) or post-mortem damage (no C/T or A/G mutations) were specifically used to estimate the haplogroup (See Supplementary Data File 4)

Pseudohaploid calls

SNP calling was conducted using an in-house custom script and Samtools. During a base quality score recalibration (BQSR) step, the quality scores of 5´ T and 3´ A in the terminal positions (10 bases) of the reads were lowered to a phred-score of 2 (#). Reads with a mapping quality ≥30 that were covering the 1240K panel positions were extracted from the BAM files and stacked with Samtools mpileup. For each of the 1240K positions, a single nucleotide with

a base quality ≥ 30 was drawn and represented as a homozygous call for that position. Transitions were treated as missing data, while SNPs with additional alleles or indels in ancient individuals were excluded from the dataset.

Population Genomic Analysis

For comparative data, 640 ancient individuals from the AADR repository were used[39–58] and along with 426 modern genomes[59–61], using Plink[62], a final dataset was constructed (see Supplementary Data Files 5 and 6). One non-European individual (Mbuti), was used as an outgroup for calculating f-statistic based tests. Along with the 30 individuals from this study, this formed a final dataset of 1096 pseudo-haploid individuals covering 1,232,975 SNPs, with a genotyping rate of ~0.60.

Principal Component Analysis

To address the low coverage of the ancient DNA individuals, lsq projection (SmartPCA, lsq projection) from the EIGENSOFT package v. 8.0.0[63,64] was applied, utilizing the high-coverage modern-day individuals to define principal components, onto which the ancient individuals were projected. One modern individual, Mbuti, was removed from the dataset and the following parameters were set to nondefault values: altnormstyle: NO, numoutlieriter: 0, killr2: NO, numoutlierevec: 0, lsqproject: Yes, and shrinkmode: Yes. The results were visualized using RStudio[65,66].

Testing for admixture

Before running admixture analysis, two low coverage individuals reported in this study (cfa006 and khf001) were removed from the dataset as well as the Mbuti individual, generating a dataset with a total genotyping rate of ~0.61 and 1093 individuals after which the software tool ADMIXTURE[67] was used assuming between 2 and 10 ancestry components (K) and 20 iterations each. Using Pong[68], the major modes over the 20 iterations per K were selected and the Admixture plots were visualised using an in-house R-script (Supplementary Figure 3). Cross-validation error estimates were used to find the best number of ancestry components (Ks) describing the data. (Supplementary Figure 4)

Kinship estimate

To assess relatedness and potential kinship among individuals we employed READ2 [69,70] for all individuals from the British Isles per archaeological period (Neolithic and Bronze Age). The lowest coverage individuals from this study (cfa006 and khf001) were excluded from the

READ2 analysis. We first divided individuals into Neolithic and post-Neolithic subsets to ensure a stable p_0 baseline within each temporal group. Pair lists are provided in Supplementary Figure 1, with proposed relationships between individuals from this paper as well as comparative data available in Supplementary Data Files 9-10. All lower-coverage pairs are shown for completeness, but are not used for interpretation.

At Monkton-up-Wimborne (Down Farm, Dorset; Neolithic; ~3350–3100 cal BCE), READ2 identifies four individuals (muw001, muw002, muw003, muw004) linked by first-degree relationships. Pairwise comparisons show muw001–muw002 and muw001–muw004 as first-degree siblings (with ≥40,000 overlapping SNPs), and the remaining MUW pairings fall within the first-degree range as well. All four individuals share the same mtDNA haplogroup (K1a), consistent with a shared maternal lineage. The two males (muw001 and muw002) are also assigned to the same Y-haplogroup (I2a2a), consistent with a shared paternal lineage. Radiocarbon midpoints overlap closely, and osteological assessment identified one adult female and three subadults; however, the available age-at-death information is insufficient to establish directionality for the remaining first-degree relationships (sibling versus parent–offspring). We therefore interpret the MUW individuals as a first-degree kin cluster with shared maternal ancestry and, for the two males, a shared paternal lineage, without specifying the generational structure of the unresolved relationships.

In the post-Neolithic subset, two relationships exceed our reporting threshold. First, cfa001 (Canada Farm, Dorset; mtDNA HV0+195; Y-haplogroup R1b1a1a2; ~2381 cal BCE) and I5379[41] (Canada Farm; mtDNA HV0+195; Y-haplogroup R1b1a1b1a1a2c1; ~2381 cal BCE) are classified as "Identical Twins/Same Individual" with 28,668 overlapping SNPs. Given identical mtDNA, date, and location, the most parsimonious interpretation is duplicate sampling of the same individual. Second, dfb002 (Fir Tree Field Pond Barrow, Dorset; mtDNA I1a1; Y-haplogroup R1b1a1a2a1a2; ~1868 cal BCE) and dfb004 (same site; mtDNA U5b2a4; Y-haplogroup R1b1a1a2a1a2; ~1878 cal BCE) form a third-degree pair supported by 39,862 overlapping SNPs, with a shared Y lineage but different maternal lines consistent with a distant paternal relative.

f3 / f4 statistics

F-statistics were conducted using the software POPSTATS[71]. Using the 28 pseudohaploidized individuals with a coverage above 0.02x, we conducted f3-outgroup statistics (Mbuti; Target1, Target2) over all pairwise comparisons, including each individual against itself for a complete square matrix (784 comparisons). An outgroup with no expected gene flow (Mbuti from SGDP)

was utilized to assess the genetic affinity among ancient British individuals (Target 1, Target 2). For visualizing the f3-outgroup matrix we used the R package pheatmap[72] with the "complete" clustering method. The f4 tests were conducted on the same 28 individuals as the f3-outgroup analyses from this study plus all comparative Neolithic and Bronze age individuals from the British Isles (see Supplementary Data File 5 and 6), and were designed as W, X; Y, Z, where the Mbuti population served as the outgroup (W), X represented the test individual, and Y and Z were assigned the two potential source populations. Specifically, the Anatolian Neolithic Farmer (ANF) population was assigned as Y, while the Yamnaya population served as Z. A deviation from zero in the f4 statistics would reject the proposed tree-like population history. Positive values suggest gene influx from ANF into the test individual, while negative values imply Pontic-steppe related gene flow. Z scores above 3 or below -3 are considered significant, indicating rejection of the proposed population tree.

qpAdm dataset construction

We assembled a new Britain-specific panel [41,46,47,51,57,73–75] and after dividing the individuals into Neolithic and post-Neolithic groups, we applied qpAdm [76–79] to all individuals with ≥30,000 overlapping SNPs.

Ancestry modelling

British Neolithic individuals are well described by a two-source model involving Early European Farmer–related ancestry (LBK_Stuttgart[54]) and Western Hunter-Gatherer ancestry (Loschbour[54]). Individuals dating after ~2550 BCE require an additional Steppe-related source and were modelled using a Yamnaya group[48,49,57,78] (see Supplementary Data Files 7-8).

qpWave

We evaluated source distinguishability with qpWave (AdmixTools2[76]), using allsnps = TRUE and block-jackknife support [77–79]. Left sets: (i) two-way—LBK_Stuttgart, Loschbour; (ii) three-way—Yamnaya, LBK_Stuttgart, Loschbour. Right (outgroups): Mbuti.DG[60,80,81] (n=2, present-day), Ethiopia_4500BP.SG[82], Papuan[61] (n=3, present-day), Georgia_Kotias.SG[56], Belgium_UP_GoyetQ116_1[52], Czech_Vestonice16[52], Italy_North_Villabruna_HG[52], Russia_MA1_HG.SG[83], Russia_Ust_Ishim_HG.DG[52], Israel_Natufian[84] (n=4), Russia_Karelia_HG[48,78], Sweden_Mesolithic.SG (SF12)[24]. The panel comprises ~1,094,206 SNPs; with allsnps=TRUE the SNP set varies by f_4-statistic. Two-way (LBK_Stuttgart, Loschbour): rank-0 test df=11, χ^2=1113, p=8.99×10^{-232}. Rank-0 is rejected, indicating the two sources are not cladal with respect to the outgroups and thus satisfy qpAdm identifiability for a two-source model (see Supplementary Data File 7).

Three-way (Yamnaya, LBK_Stuttgart, Loschbour): rank-0 df=22, χ^2=1931, p≈0; rank-1 df=10, χ^2=406, p=4.79×10^{-81}; nested $\Delta\chi^2$=1524, p=1.98×10^{-319}. Both rank-0 and rank-1 are rejected, implying at least two independent ancestry streams among the three sources and meeting qpAdm identifiability for a three-source model separating Steppe-, Farmer-, and WHG-related ancestry (see Supplementary Data File 8).

Trend tests

We assessed whether qpAdm-derived ancestry proportions covary with time and geography using ordinary least squares (OLS) linear regression. For each outcome (LBK_Stuttgart and Loschbour; and Yamnaya where applicable), we fit a fixed set of univariate models (Age, Longitude, Latitude) and bivariate models involving at most two predictors (including their interaction): additive models (Age+Longitude, Age+Latitude, Longitude+Latitude) and two-way interaction models (Age×Longitude, Age×Latitude, Longitude×Latitude).

To separate Neolithic and Bronze Age contexts, we applied a conservative temporal cutoff of 2550–800 cal BCE for the Bronze Age[85,86]**.** All earlier individuals carrying a farmer-related component were assigned to the Neolithic subset. Two Scottish Neolithic individuals with calibrated dates marginally younger than the 2550 BCE threshold (I2932: 2460 cal BCE; I2977: 2540 cal BCE) were nevertheless retained in the Neolithic subset based on their ancestry profiles, bringing the Neolithic sample size to n = 166. Analyses were run separately for the Neolithic (n = 166) and Bronze Age (n = 163) subsets and are reported in Supplementary Data File 11.

In the Neolithic subset, qpAdm ancestry variation is dominated by geography rather than time (Supplementary Figure 10). Across Britain during the Neolithic, LBK_Stuttgart shows a significant north–south cline, decreasing with latitude (β = −0.00390 ± 0.00125, P = 0.00214; R^2 = 0.056), while Loschbour shows the opposite latitudinal trend (β = +0.00390 ± 0.00125, P = 0.00214; R^2 = 0.056). A simple additive spatial model improves fit (Longitude + Latitude: R^2 = 0.0779; P (overall F-test) = 0.00134), and the Longitude × Latitude model provides the highest bivariate fit (R^2 = 0.136; P (overall F-test) = 2.89 × 10^{-5}), suggesting spatial heterogeneity in ancestry gradients.

In the Bronze Age subset (n = 163), the main signal is temporal: LBK_Stuttgart increases toward the present (Age-only: β_{Age} = −3.97×10^{-5} ± 1.41×10^{-5} per BCE year; P = 0.00545; R^2 = 0.047; negative β indicates increase toward the present because BCE decreases forward

in time), while Yamnaya shows the opposite direction ($\beta\_Age = +3.98\times10^{-5} \pm 1.43\times10^{-5}$ per BCE year; $P = 0.00602$; $R^2 = 0.046$). This direction matches previously reported temporal shifts in Bronze Age ancestry profiles [41,57]. Loschbour shows no detectable temporal trend ($\beta\_Age = 2.10\times10^{-7} \pm 4.77\times10^{-6}$; $P = 0.965$; $R^2 \approx 0$). These age effects remain in additive models including latitude (Age+Latitude: LBK_Stuttgart $\beta\_Age = -3.93\times10^{-5} \pm 1.41\times10^{-5}$, $P = 0.00596$; Yamnaya $\beta\_Age = +3.93\times10^{-5} \pm 1.43\times10^{-5}$, $P = 0.00662$), with no strong standalone spatial clines in univariate longitude/latitude models. Adding an Age×Latitude term improves model fit for both LBK_Stuttgart and Yamnaya (Age×Latitude models: $R^2 = 0.079$–0.081; P (overall F-test) = 0.00435 and 0.00377), suggesting latitude-dependent variation in the temporal trend.

When restricting analyses to individuals generated in this study, the Neolithic Loschbour–latitude association is not detectable ($n = 10$; $\beta\_Lat = 0.0561 \pm 0.0497$ per degree latitude; $P = 0.291$), and the Bronze Age temporal associations are likewise not detectable ($n = 11$; LBK_Stuttgart $\beta\_Age = +7.83 \times 10^{-5} \pm 7.65 \times 10^{-5}$ per BCE year, $P = 0.333$; Yamnaya $\beta\_Age = -1.48 \times 10^{-5} \pm 9.09 \times 10^{-5}$ per BCE year, $P = 0.875$), consistent with limited power and subset-specific sampling.

We also compared the distribution of LBK-Stuttgart related ancestry between individuals from this study and the comparative dataset using two-sided t-tests, run separately for the Neolithic and Bronze Age subsets and for two geographic panels (British Isles and England only) (Supplementary Figure 14).

In addition, we assessed whether any single individual shows an unusual qpAdm ancestry proportion relative to the rest of the dataset using an empirical resampling (“individual-vs-rest”) test (Supplementary Data File 12). For each individual and ancestry component, we compared the observed proportion to the remaining individuals and computed a two-sided empirical P-value by repeatedly sampling one value with replacement from that comparison pool.

In the Neolithic subset, no individuals were significant after Benjamini–Hochberg correction for either LBK_Stuttgart or Loschbour. In the Bronze Age subset, results were likewise largely non-significant after Benjamini–Hochberg correction; the only Benjamini–Hochberg-significant deviation was for one individual in the Loschbour component (muw005), and this outlier signal should be treated as descriptive because the test ignores qpAdm standard errors.

Testing for retained local Neolithic ancestry after Bell Beaker-associated migration

To test whether Bronze Age individuals from this study could be modelled as fully captured by incoming continental Bell Beaker-related ancestry, or whether they retained additional ancestry specifically related to local British Neolithic farmers, we fitted two-way qpAdm models of the form Target = Local Neolithic + Bell Beaker Germany (Supplementary Data File 13). The Local Neolithic source comprised nine Neolithic individuals from this study with >50,000 SNPs: che001, muw001, muw002, muw004, slb003, slb004, wrb002, wrb004 and wrb006. The continental source comprised ten Bell Beaker-associated individuals from Germany: I6590, I6591, I5525, I5519, I5833, I3601, I5835, I6624, I3594 and I5531[41], used as a proxy for incoming continental Bell Beaker ancestry.

Bronze Age targets were retained using a minimum threshold of 30,000 SNPs. Models were run with a fixed right set including Mbuti [60,80,81](n=2, present-day), Papuan [61](n=3, present-day), Georgia_Kotias.SG[56], Czech_Vestonice16[52], Italy_North_Villabruna_HG[52], Russia_MA1_HG.SG[83], Russia_Ust_Ishim_HG.DG[34], Israel_Natufian[54] (n=4), Yamnaya[48,49,57,78] (n=8) and Sweden_Mesolithic.SG[24] (SF12). Because the Bell Beaker Germany source already carries Neolithic farmer-related ancestry, the Local Neolithic coefficient was interpreted as excess ancestry related to local British Neolithic groups beyond that captured by the continental Bell Beaker proxy.

Ten Bronze Age individuals from this study passed the target SNP threshold. Overall, they showed a predominantly Bell Beaker Germany-like ancestry profile (Supplementary Figure 15). Most individuals had Local Neolithic coefficients estimated at zero or with 95% confidence intervals overlapping zero, providing no robust evidence for additional local Neolithic-related ancestry beyond that already captured by the Bell Beaker Germany source. Chronologically, the earliest individual, lot001, was estimated as entirely Bell Beaker Germany-related in this model. The following two early individuals, cfa001 and hdw002, were compatible with two-way models including a Local Neolithic component, but their Local Neolithic confidence intervals overlapped zero and therefore do not provide statistically robust evidence for this ancestry. Later individuals were again mostly estimated as entirely or nearly entirely Bell Beaker Germany-related. The clearest exception was dfr001 from Fir Tree Field Ring Ditch, which showed a positive Local Neolithic estimate of 45.3 ± 20.9%, with a 95% confidence interval of 4.2–86.3%; this model was feasible and non-rejected ($p = 0.448$). However, dfr001 had fewer than 50,000 SNPs, whereas samples above this threshold are indicated with bold labels in the accompanying plot.

We then applied the same two-way qpAdm framework to all available English Bronze Age comparative individuals passing the 30,000 SNP target threshold (n=118; Supplementary Figure 16). This broader comparison showed the same general pattern: most English Bronze Age individuals were modelled as entirely or near-entirely Bell Beaker Germany-related, but a minority were compatible with two-way models including Local Neolithic ancestry.

Overall, the tested Bronze Age individuals show a predominantly continental Bell Beaker-related ancestry profile, with limited evidence for retained local Neolithic-related ancestry in some individuals or regional pockets. Although the age-ordered results are compatible with a decline of detectable local Neolithic-related ancestry through time, this pattern remains tentative because several estimates are imprecise, many confidence intervals overlap zero, and some informative individuals have relatively low SNP counts.

Radiocarbon dating

The radiocarbon dating was conducted at the National Laboratory for Age Determination (TRa), Trondheim, Norway; Oxford AMS (OxA); Scottish Universities Environmental Research Centre (SUERC), Glasgow, Scotland; 14CHRONO Centre for Climate, the Environment and Chronology (UBA), Belfast, United Kingdom, Waikato Radiocarbon Dating Laboratory, University of Waikato, New Zealand (NZA), British Museum (BM), London, United Kingdom and Ångström Laboratory Tandem Laboratory, Uppsala University (Ua). The calibration of the radiocarbon dates was conducted using the OxCal[87] online software version 4.4 and the IntCal20 atmospheric curve[88]

Map Plotting

A zoomed-in map of southwestern England, showing the location of each archaeological site analysed, was created in RStudio[65,66,89]. To minimize point overlap, a jittering effect was applied to the spatial coordinates with a factor of 0.05. The map data includes information from Ordnance Survey, the Office of National Statistics, National Records of Scotland, and the Land and Property Services of Northern Ireland. Data is © Crown copyright and database right, 2016, and is licensed under the Open Government Licence (http://www.nationalarchives.gov.uk/doc/open-government-licence/version/3)

Supplementary Figures

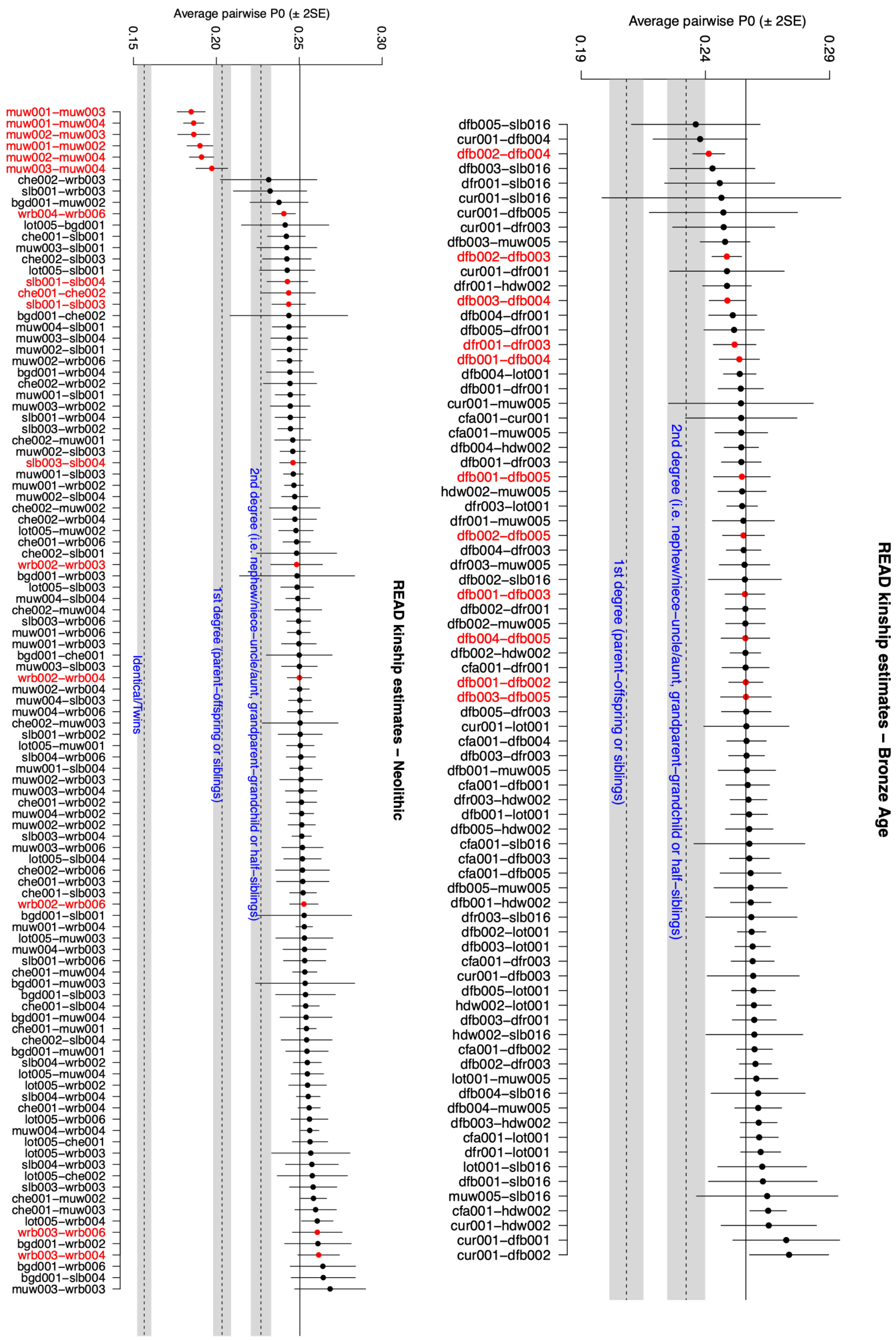

Supplementary Figure 1. READ2 results for the Neolithic and Bronze Age groups. Within burial site comparisons are highlighted in red.

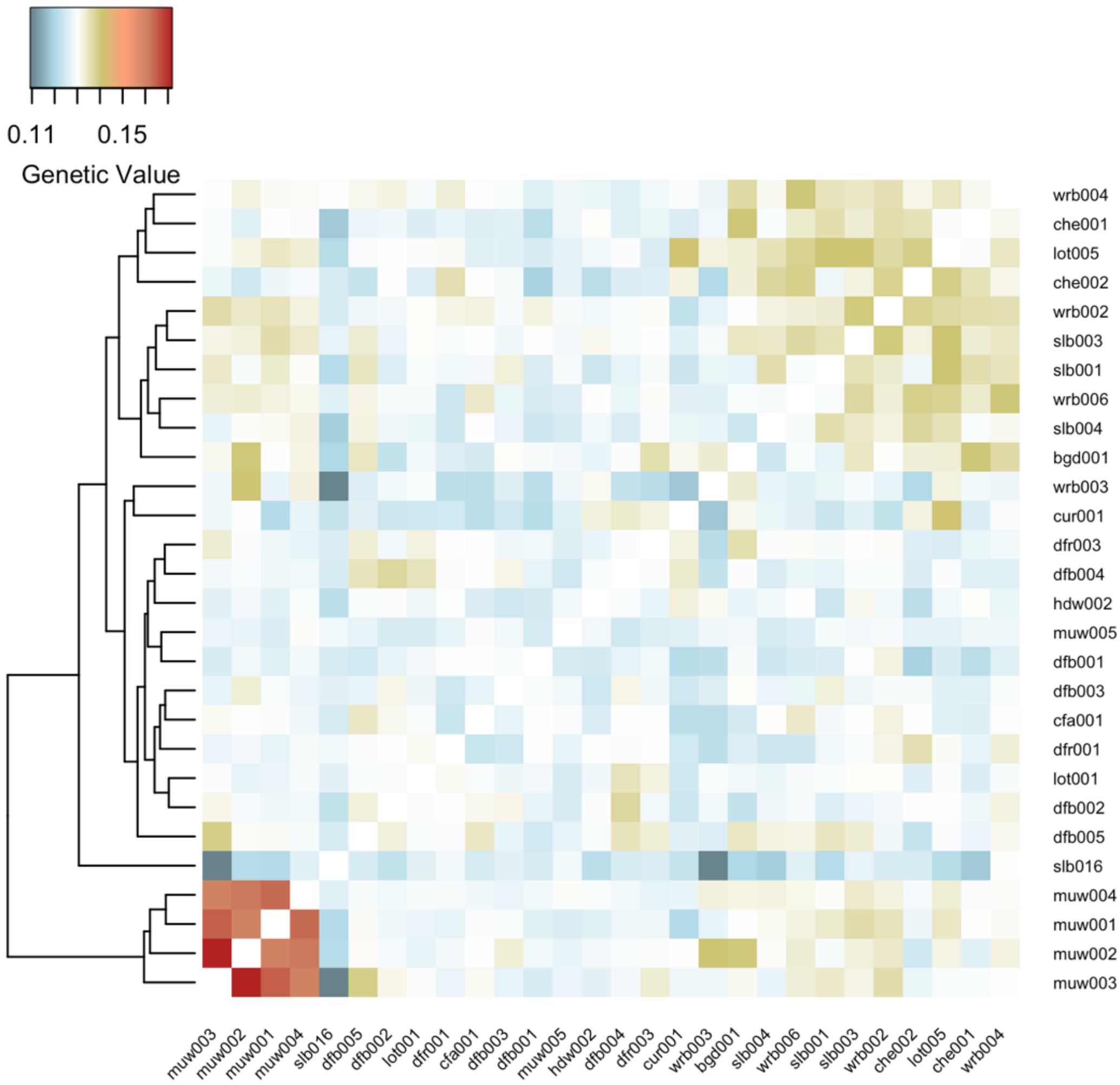

Supplementary Figure 2. Pairwise f3-outgroup (Target1; Target2; Outgroup (*Mbuti*)) heatmap plot for the individuals in this study. Two individuals (khf001 and cfa006) were not used in the f4 analysis due to their low coverage.

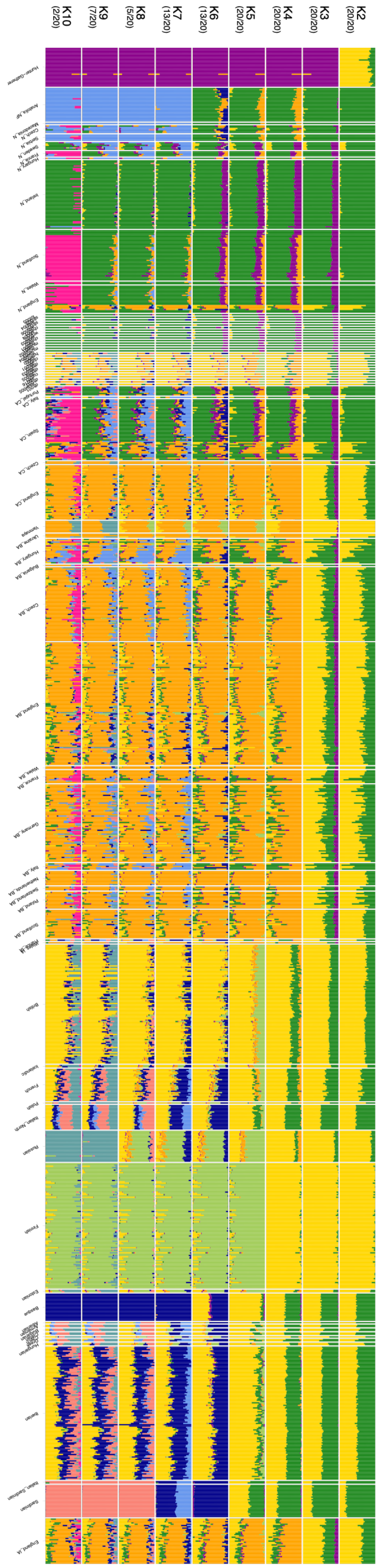

Supplementary Figure 3. ADMIXTURE plot for the entire dataset (K2 - K10, 20 iterations)

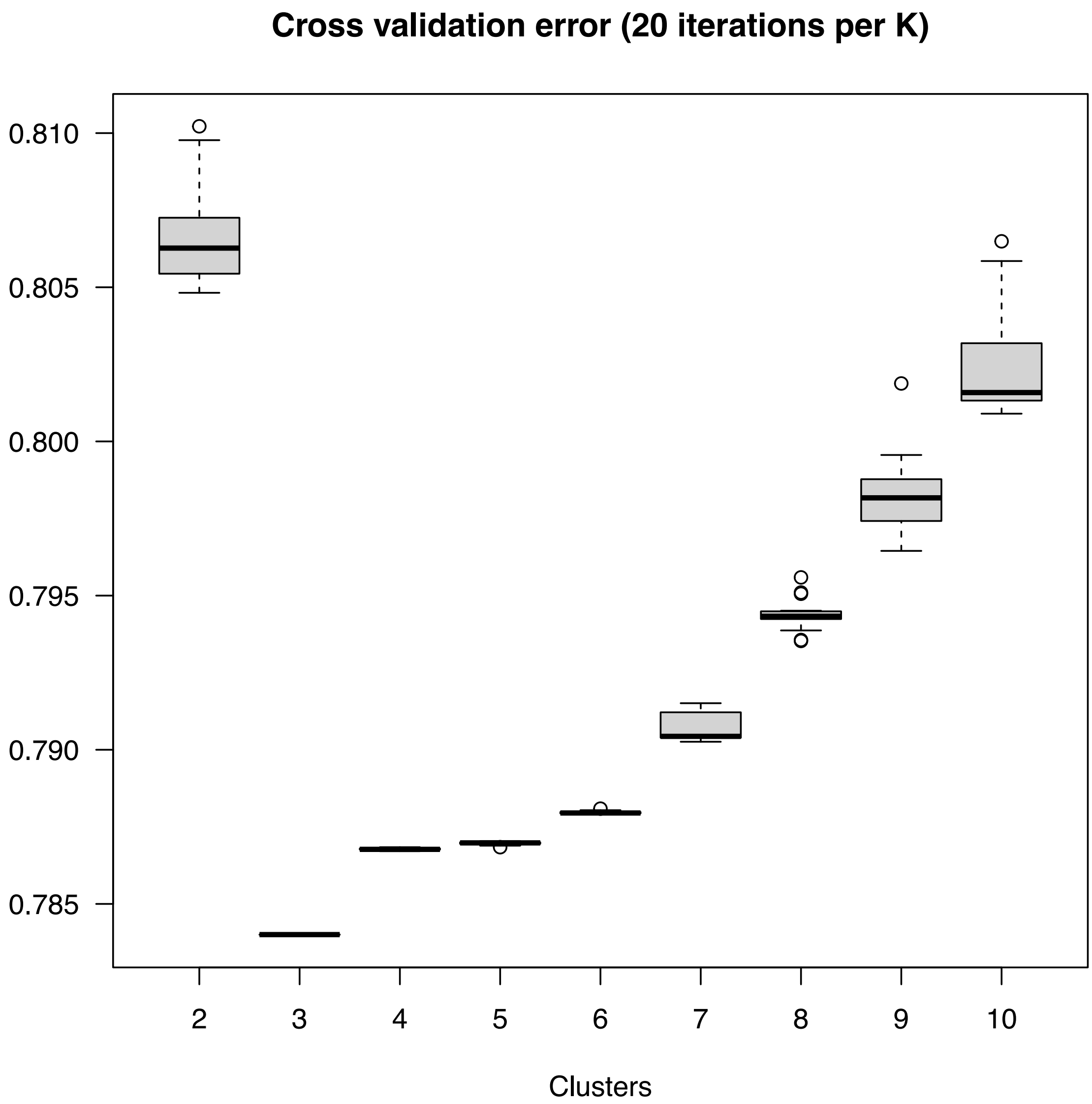

Supplementary Figure 4. Cross-validation scores over 20 iterations of Admixture analyses.

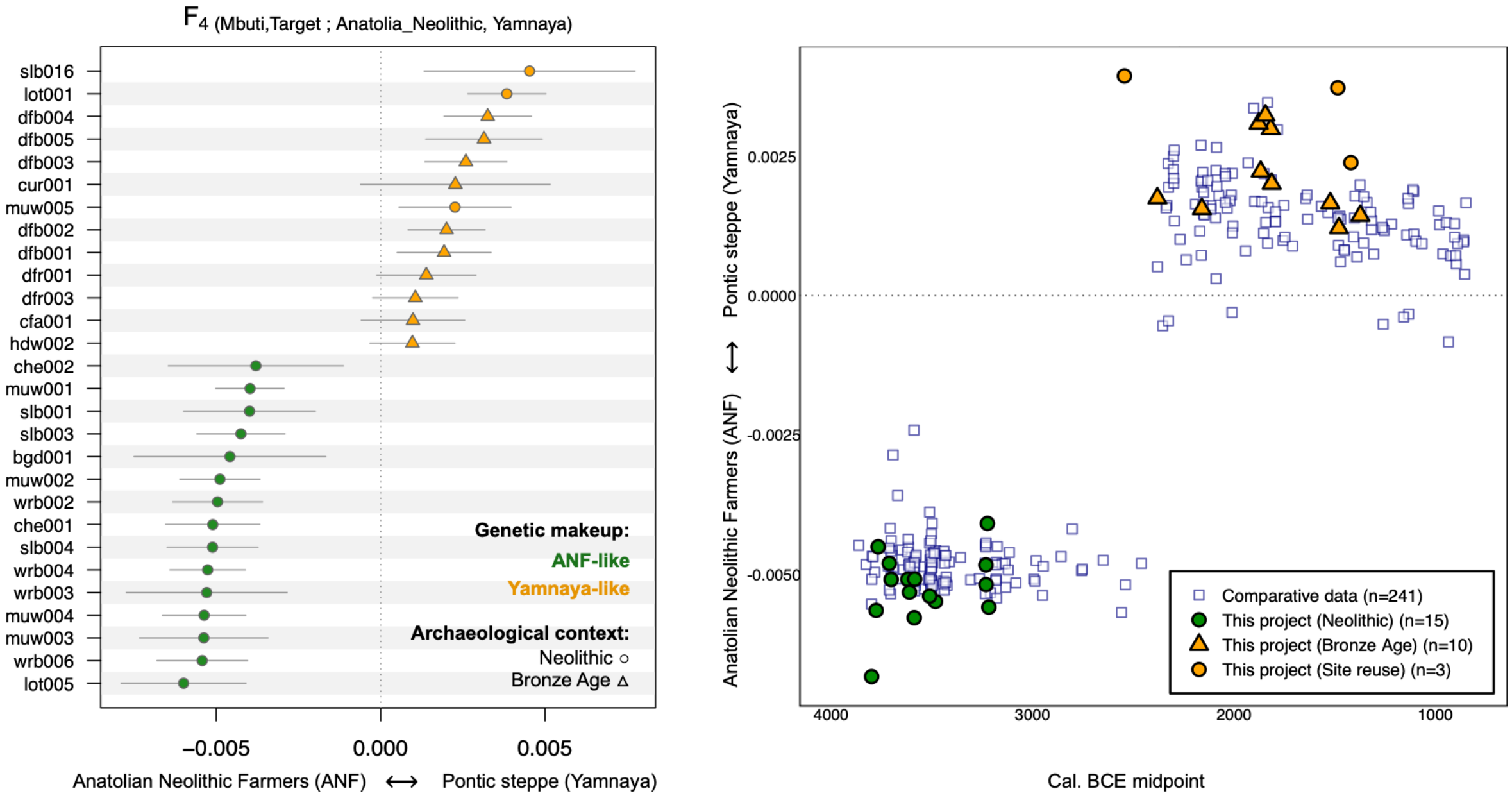

Supplementary Figure 5. Genetic affinity of the individuals from this study, between ANF and Pontic steppe Yamnaya–associated individuals using f4-statistics (Mbuti, test individual from this study; ANF, Yamnaya). Individuals are ordered according to (calibrated) radiocarbon-date, with the oldest individual in the bottom. Individual symbols correspond to archaeological context while colour corresponds to genetic affinity. Right panel, Genetic affinity between ANF and Pontic steppe Yamnaya–associated individuals for ancient British Isles individuals (this study and comparative datasets), restricted to individuals that passed quality control (PASS), showed no evidence of contamination, and had directly dated radiocarbon measurements.

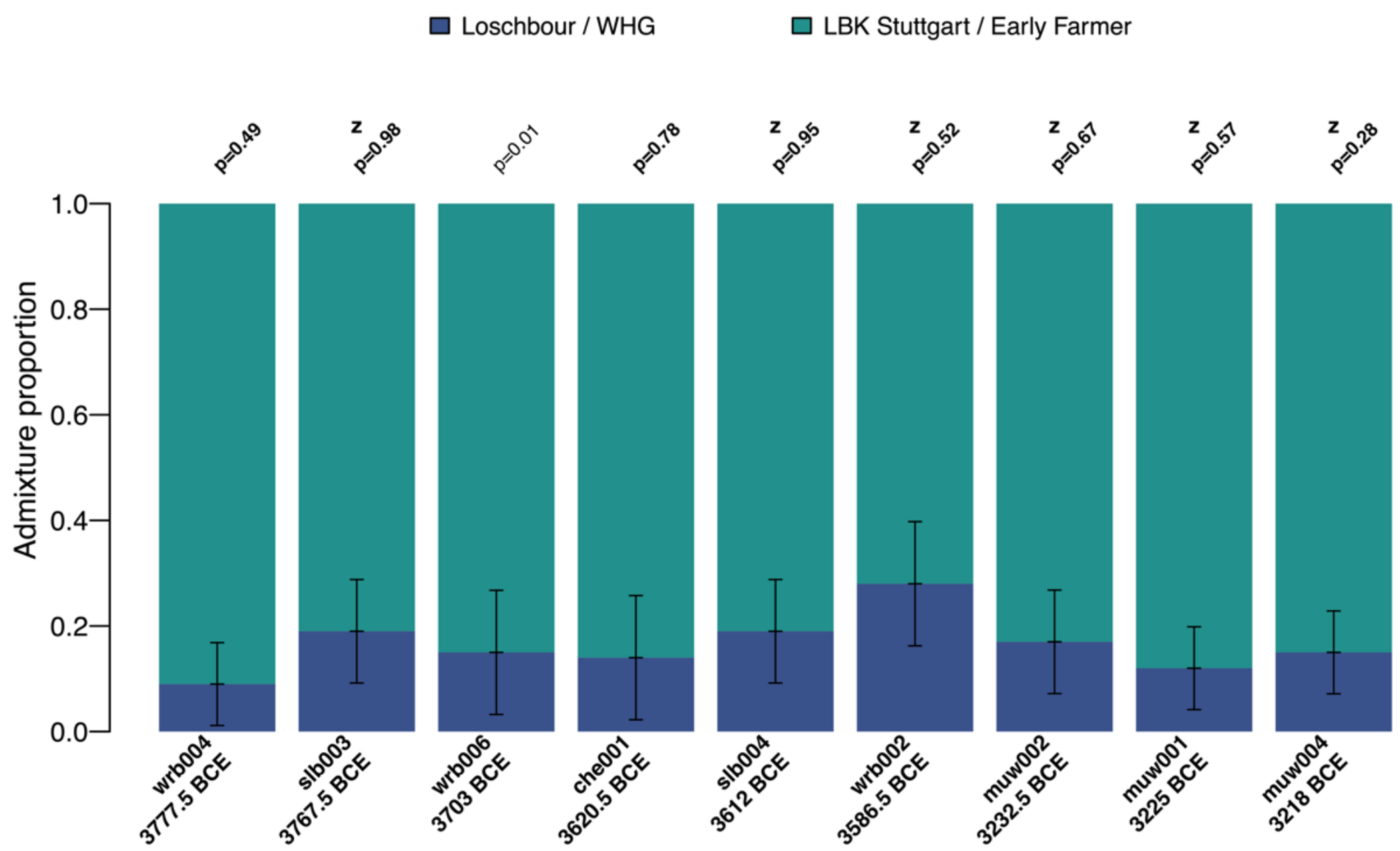

Supplementary Figure 6. Two-way qpAdm estimates for focal individuals from this paper, modelled as a mixture of Early Farmer–related ancestry (LBK_Stuttgart; green) and Western Hunter-Gatherer ancestry (Loschbour; blue). Bars show ancestry proportions with 95% confidence intervals anchored at the left edge of the LBK_Stuttgart segment and the right edge of the Loschbour segment. All individuals pass a ≥30,000 SNP cutoff (bold labels: >50,000 SNPs); "z" marks indicate |z| ≥ 3 for the chosen source (Loschbour).

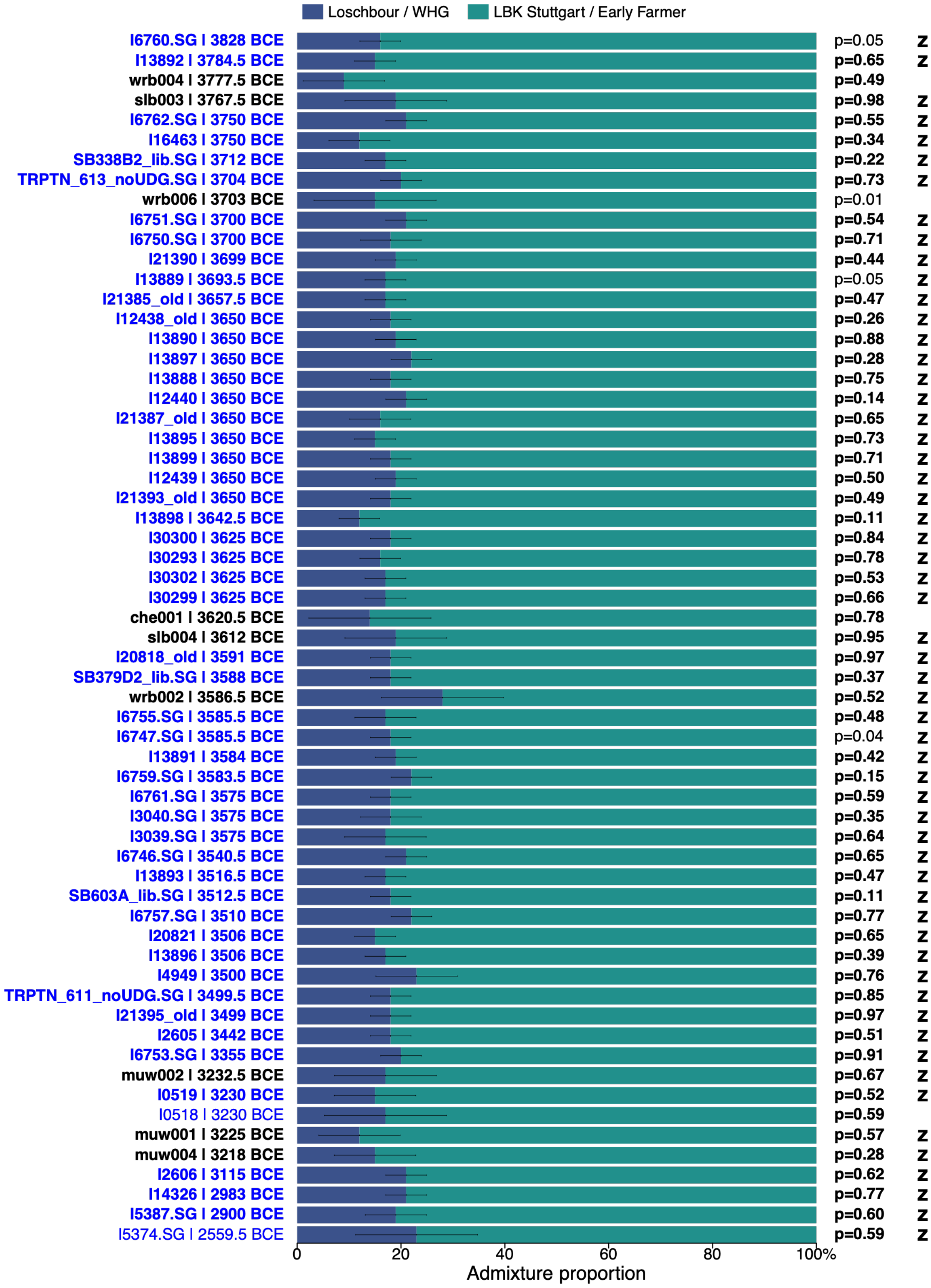

Supplementary Figure 7. qpAdm estimates for the two-way model (LBK_Stuttgart + Loschbour), including comparative Neolithic individuals from England (blue labels).

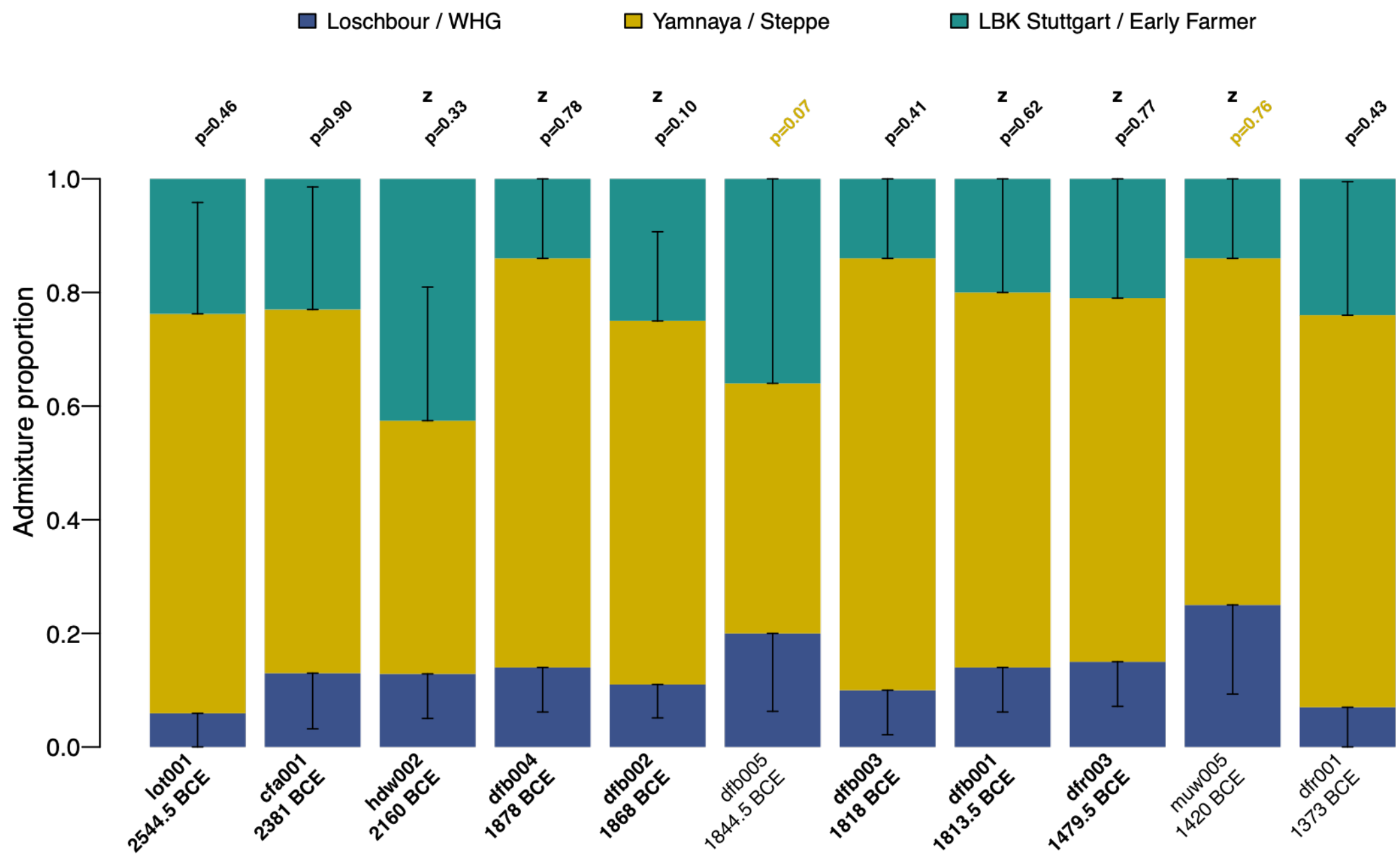

Supplementary Figure 8. Three-way qpAdm estimates for focal West Country individuals, modelled as contributions from Early Farmer–related ancestry (LBK_Stuttgart; green), Western Hunter-Gatherer–related ancestry (Loschbour; blue), and Steppe-related ancestry (Yamnaya; gold). Bars show ancestry proportions ordered from older to younger individuals, left to right. Confidence intervals are shown for the Loschbour and LBK_Stuttgart components. Sample labels are bold where the model includes >50,000 SNPs; all plotted individuals pass the ≥30,000 SNP cutoff. P-values are coloured when the Yamnaya confidence interval crosses zero, indicating uncertainty in the Steppe-related component. A "z" marker indicates Loschbour z ≥ 3. Asterisks indicate non-feasible qpAdm solutions.

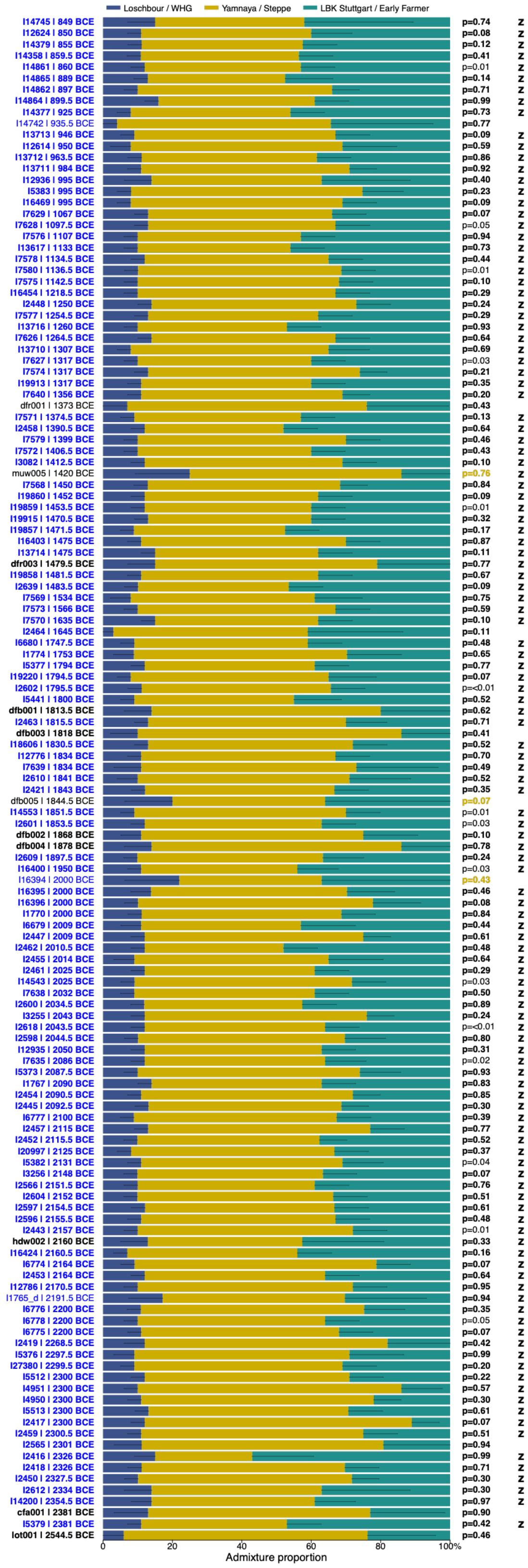

Supplementary Figure 9. qpAdm estimates for the three-way model (LBK_Stuttgart + Loschbour + Yamnaya), including comparative Bronze Age individuals from England (blue labels).

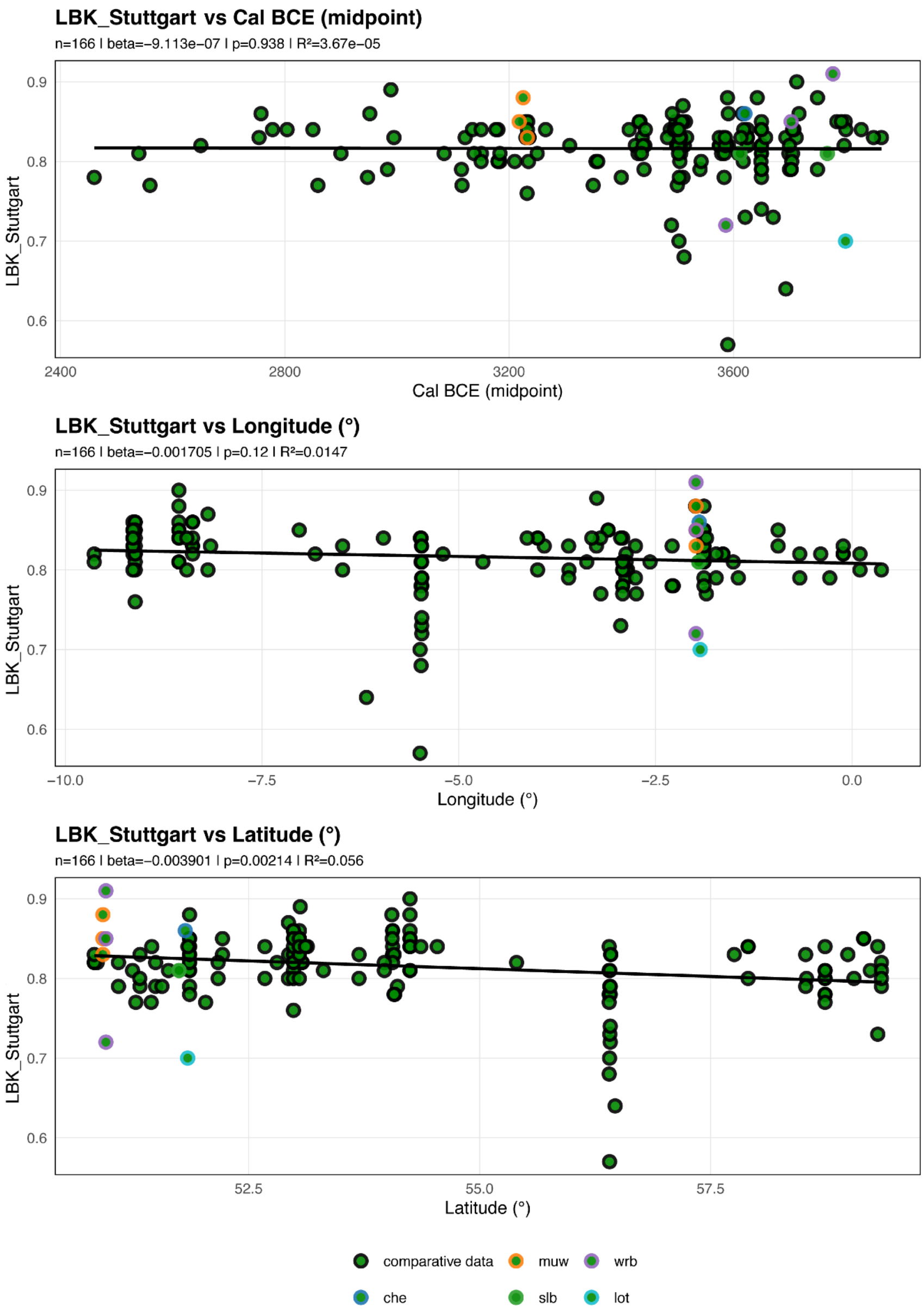

Supplementary Figure 10. Panels show qpAdm-derived ancestry proportions for the LBK-Stuttgart-related component plotted against calibrated radiocarbon age (Cal BCE midpoint), longitude, and latitude for Neolithic individuals from Britain. Points represent individuals, with individuals from this study highlighted by coloured outlines and comparative Neolithic individuals shown in black. Black lines indicate ordinary least-squares regression fits; panel subtitles report sample size (n), regression slope, p-value, and coefficient of determination (R^2). As two sources are used for modelling, the Loschbour-related component is the mirror opposite of LBK-Stuttgart.

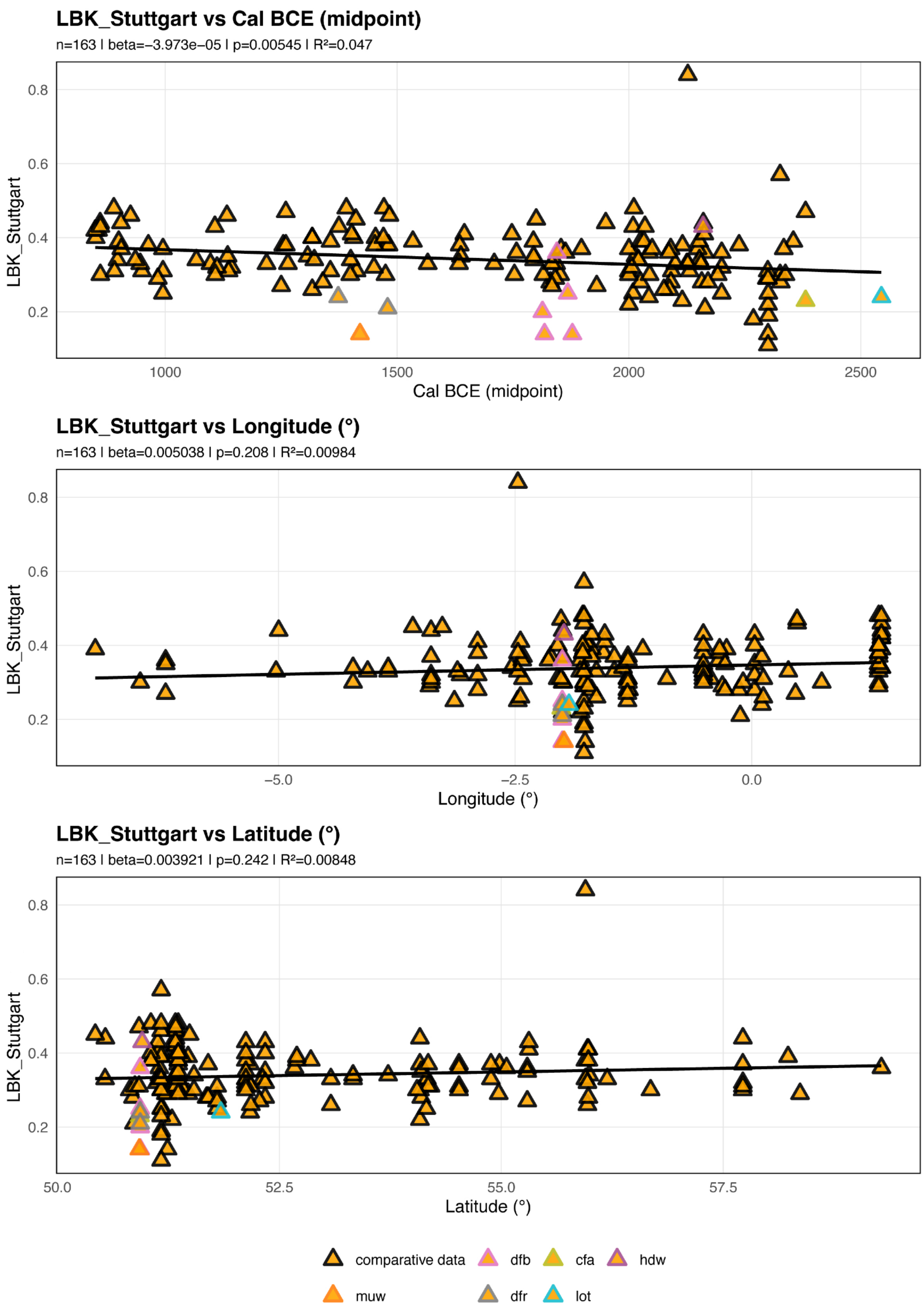

Supplementary Figure 11. Panels show qpAdm-derived ancestry proportions for the LBK-Stuttgart-related component plotted against calibrated radiocarbon age (Cal BCE midpoint), longitude, and latitude for post-Neolithic individuals from Britain. Triangles represent individuals, with individuals from this study highlighted by coloured outlines and comparative Bronze Age individuals shown in black. Black lines indicate ordinary least-squares regression fits; panel subtitles report sample size (n), regression slope, p-value, and coefficient of determination (R^2).

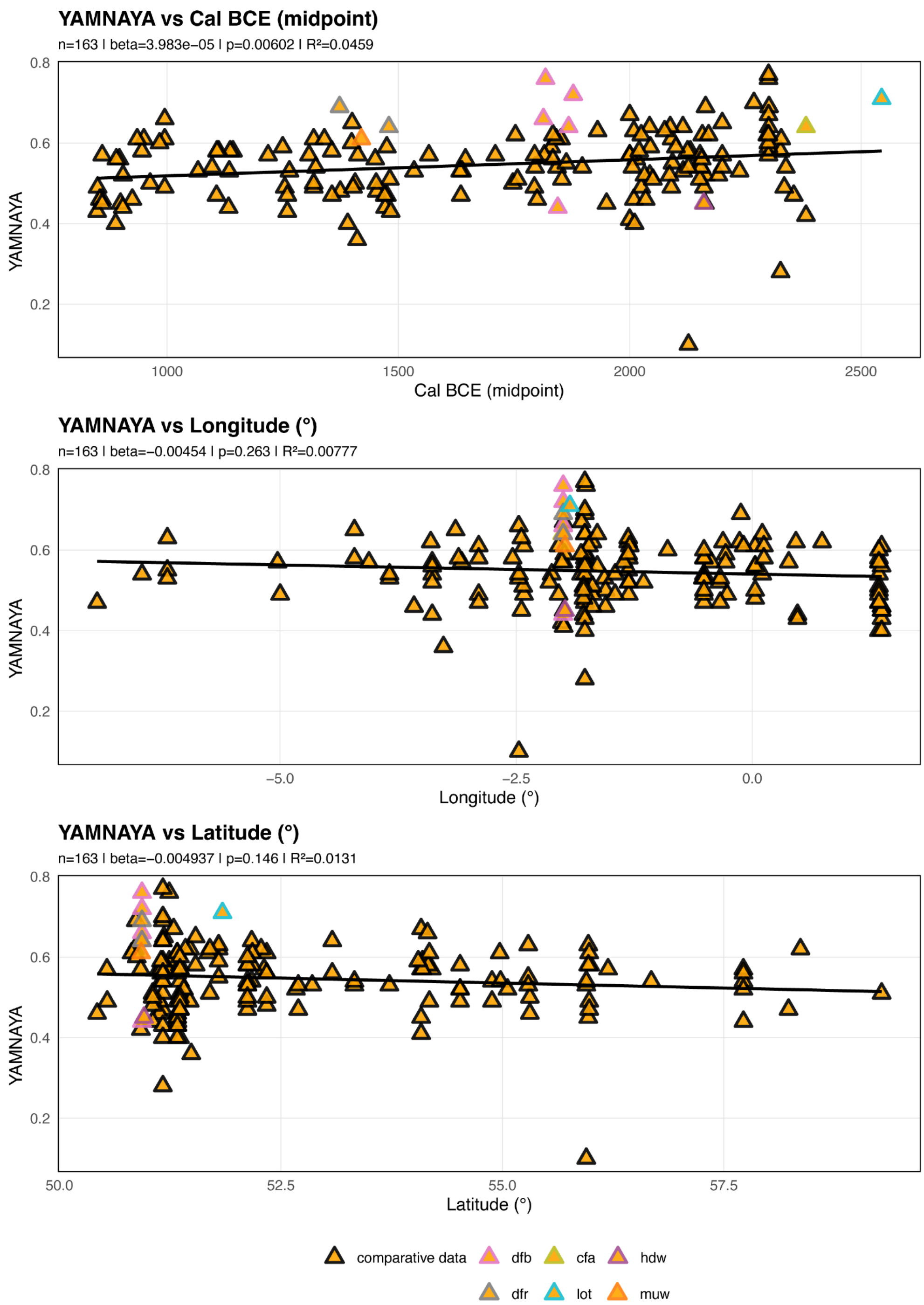

Supplementary Figure 12. Panels show qpAdm-derived ancestry proportions for the Yamnaya-related component plotted against calibrated radiocarbon age (Cal BCE midpoint), longitude, and latitude for post-Neolithic individuals from Britain. Triangles represent individuals (jittered for visibility), with individuals from this study highlighted by coloured outlines and comparative Bronze Age individuals shown in black. Black lines indicate ordinary least-squares regression fits; panel subtitles report sample size (n), regression slope, p-value, and coefficient of determination (R^2).

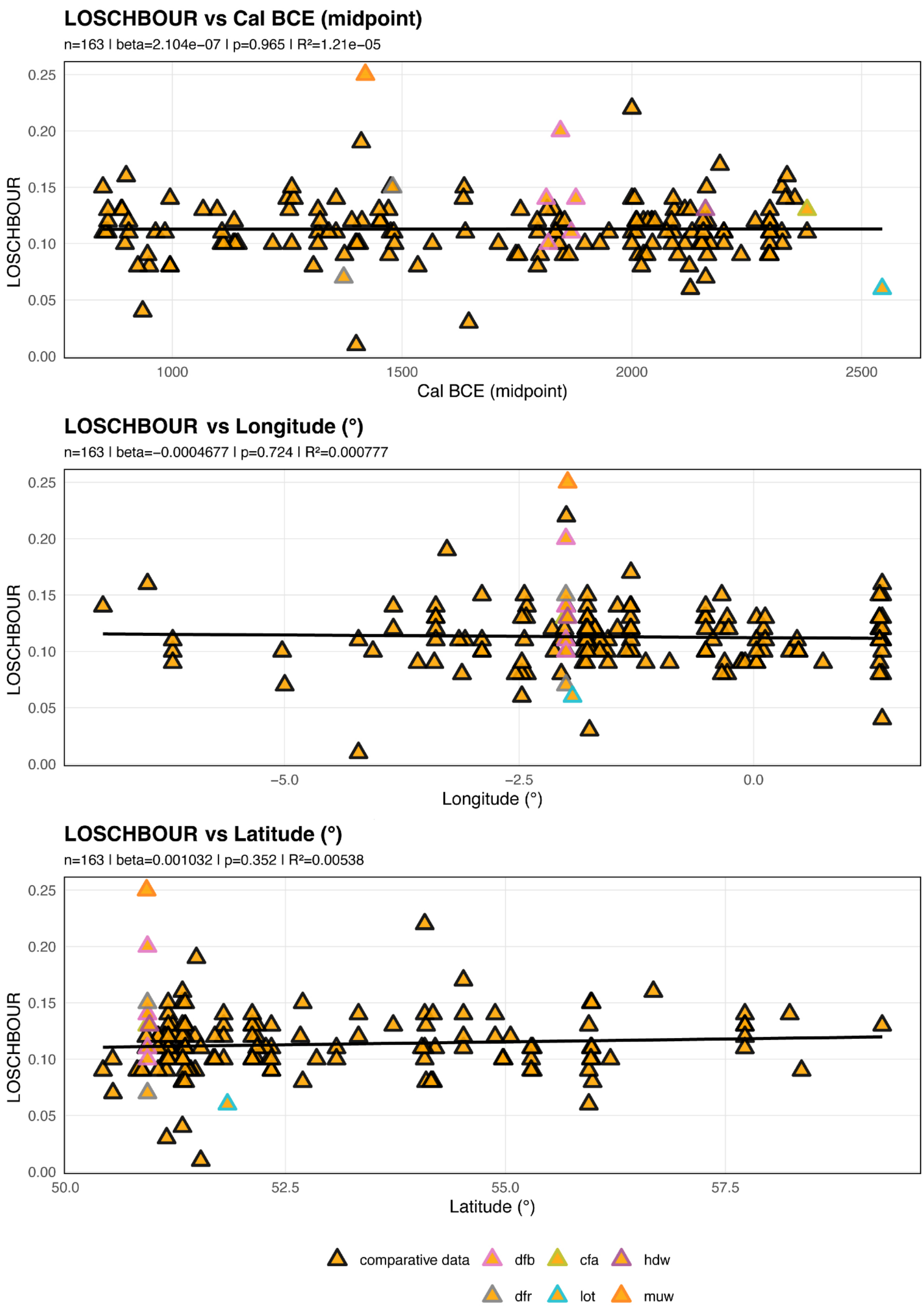

Supplementary Figure 13. Panels show qpAdm-derived ancestry proportions for the Loschbour-related component plotted against calibrated radiocarbon age (Cal BCE midpoint), longitude, and latitude for post-Neolithic individuals from Britain. Triangles represent individuals, with individuals from this study highlighted by coloured outlines and comparative Bronze Age individuals shown in black. Black lines indicate ordinary least-squares regression fits; panel subtitles report sample size (n), regression slope, p-value, and coefficient of determination (R^2).

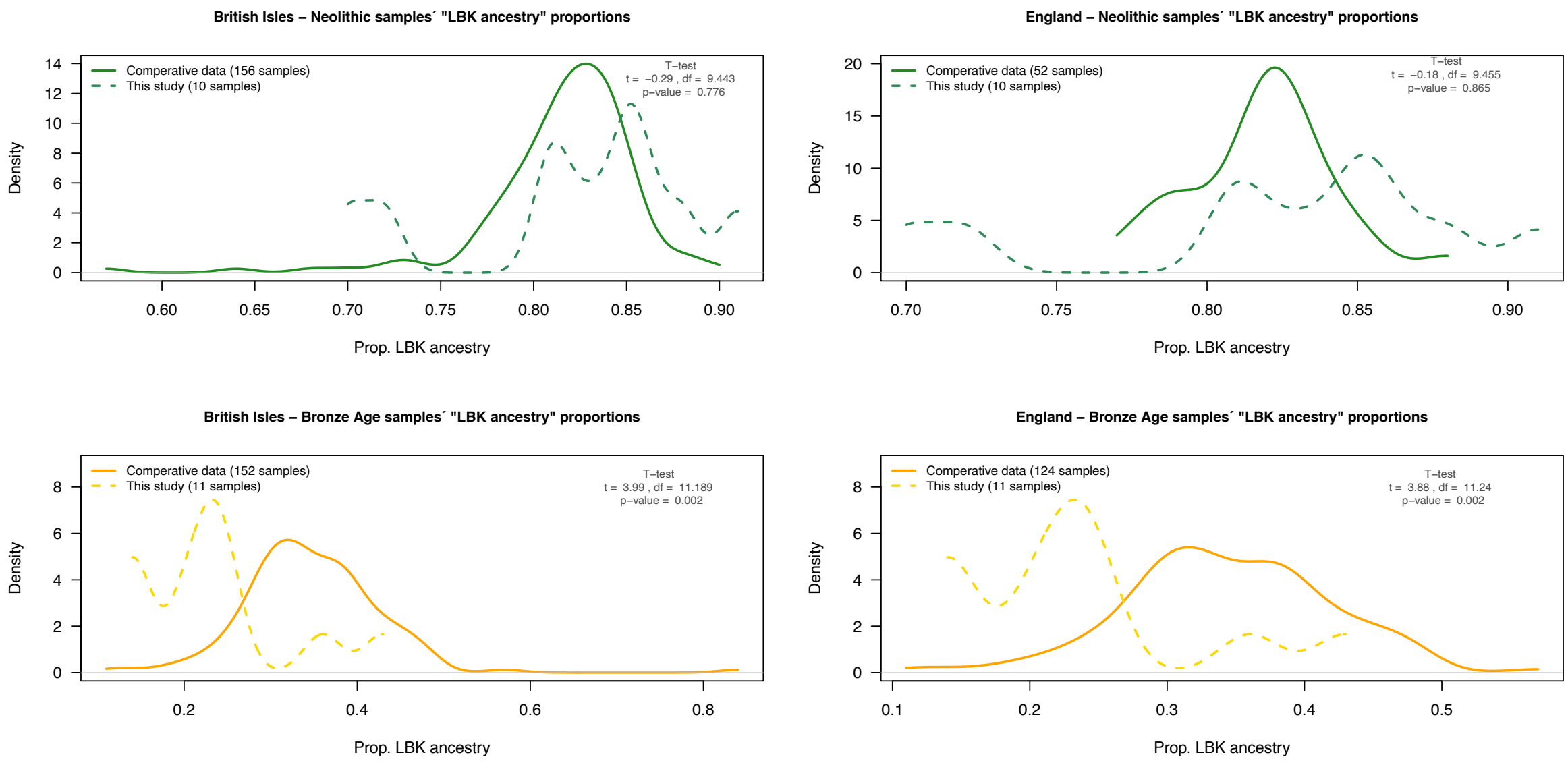

Supplementary Figure 14. Density plot of LBK-related ancestry variation across all Neolithic (top panel) and Bronze age (bottom panel) individuals across the British Isles (left panel) and only England (right panel). Comparative data are shown with solid lines while data from this study are shown with dashed lines. Differences in LBK ancestry distribution between the datasets were tested with T-tests.

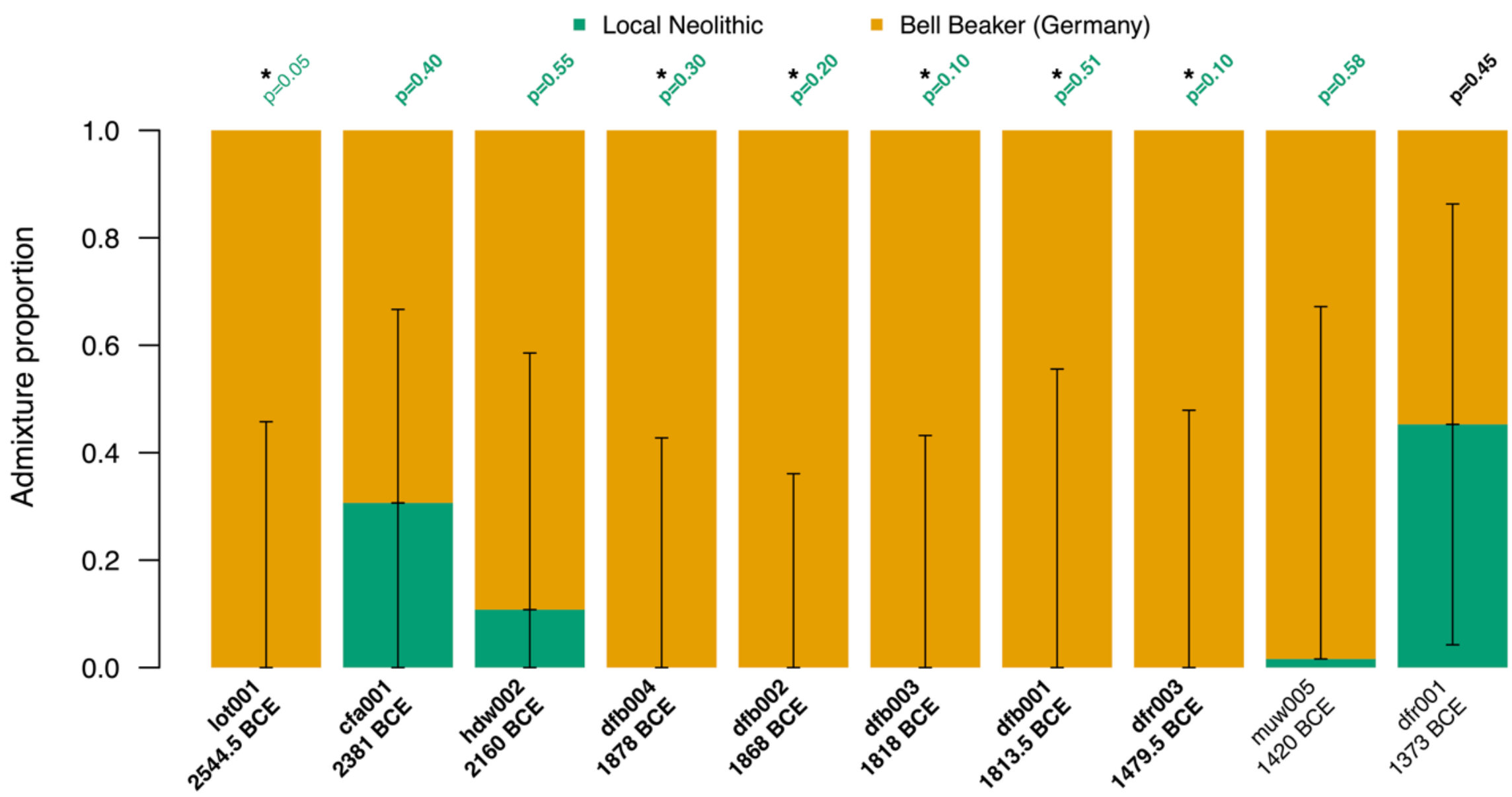

Supplementary Figure 15. Two-way qpAdm modelling of Bronze Age individuals from this study as a mixture of Local Neolithic and Bell Beaker Germany sources. Only Bronze Age targets from this study with >30,000 SNPs are shown. Samples are ordered chronologically from oldest to youngest. Bold sample labels indicate individuals with >50,000 SNPs; regular labels indicate individuals below this threshold. Bars show estimated ancestry proportions,

with Local Neolithic shown in green and Bell Beaker Germany in orange. Error bars indicate 95% confidence intervals. P-values are shown above each sample; p-values are coloured when the 95% confidence interval for the Local Neolithic source overlaps zero. Asterisks indicate models that were non-feasible under the two-way qpAdm fit.

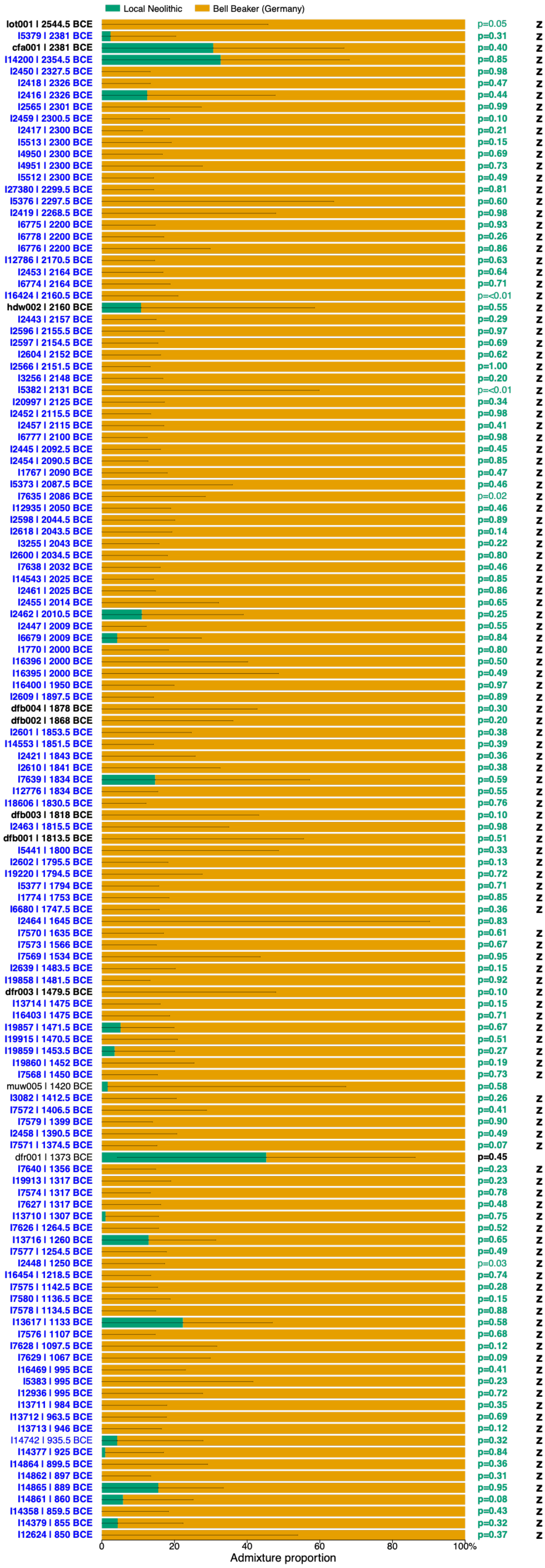

Local Neolithic
Bell Beaker (Germany)
lot001 | 2544.5 BCE p=0.05 Z
I5379 | 2381 BCE p=0.31 Z
cfa001 | 2381 BCE p=0.40 Z
I14200 | 2354.5 BCE p=0.85 Z
I2450 | 2327.5 BCE p=0.98 Z
I2418 | 2326 BCE p=0.47 Z
I2416 | 2326 BCE p=0.44 Z
I2565 | 2301 BCE p=0.99 Z
I2459 | 2300.5 BCE p=0.10 Z
I2417 | 2300 BCE p=0.21 Z
I5513 | 2300 BCE p=0.15 Z
I4950 | 2300 BCE p=0.69 Z
I4951 | 2300 BCE p=0.73 Z
I5512 | 2300 BCE p=0.49 Z
I27380 | 2299.5 BCE p=0.81 Z
I5376 | 2297.5 BCE p=0.60 Z
I2419 | 2268.5 BCE p=0.98 Z
I6775 | 2200 BCE p=0.93 Z
I6778 | 2200 BCE p=0.26 Z
I6776 | 2200 BCE p=0.86 Z
I12786 | 2170.5 BCE p=0.63 Z
I2453 | 2164 BCE p=0.64 Z
I6774 | 2164 BCE p=0.71 Z
I16424 | 2160.5 BCE p=<0.01 Z
hdw002 | 2160 BCE p=0.55 Z
I2443 | 2157 BCE p=0.29 Z
I2596 | 2155.5 BCE p=0.97 Z
I2597 | 2154.5 BCE p=0.69 Z
I2604 | 2152 BCE p=0.62 Z
I2566 | 2151.5 BCE p=1.00 Z
I3256 | 2148 BCE p=0.20 Z
I5382 | 2131 BCE p=<0.01 Z
I20997 | 2125 BCE p=0.34 Z
I2452 | 2115.5 BCE p=0.98 Z
I2457 | 2115 BCE p=0.41 Z
I6777 | 2100 BCE p=0.98 Z
I2445 | 2092.5 BCE p=0.45 Z
I2454 | 2090.5 BCE p=0.85 Z
I1767 | 2090 BCE p=0.47 Z
I5373 | 2087.5 BCE p=0.46 Z
I7635 | 2086 BCE p=0.02 Z
I12935 | 2050 BCE p=0.46 Z
I2598 | 2044.5 BCE p=0.89 Z
I2618 | 2043.5 BCE p=0.14 Z
I3255 | 2043 BCE p=0.22 Z
I2600 | 2034.5 BCE p=0.80 Z
I7638 | 2032 BCE p=0.46 Z
I14543 | 2025 BCE p=0.85 Z
I2461 | 2025 BCE p=0.86 Z
I2455 | 2014 BCE p=0.65 Z
I2462 | 2010.5 BCE p=0.25 Z
I2447 | 2009 BCE p=0.55 Z
I6679 | 2009 BCE p=0.84 Z
I1770 | 2000 BCE p=0.80 Z
I16396 | 2000 BCE p=0.50 Z
I16395 | 2000 BCE p=0.49 Z
I16400 | 1950 BCE p=0.97 Z
I2609 | 1897.5 BCE p=0.89 Z
dfb004 | 1878 BCE p=0.30 Z
dfb002 | 1868 BCE p=0.20 Z
I2601 | 1853.5 BCE p=0.38 Z
I14553 | 1851.5 BCE p=0.39 Z
I2421 | 1843 BCE p=0.36 Z
I2610 | 1841 BCE p=0.38 Z
I7639 | 1834 BCE p=0.59 Z
I12776 | 1834 BCE p=0.55 Z
I18606 | 1830.5 BCE p=0.76 Z
dfb003 | 1818 BCE p=0.10 Z
I2463 | 1815.5 BCE p=0.98 Z
dfb001 | 1813.5 BCE p=0.51 Z
I5441 | 1800 BCE p=0.33 Z
I2602 | 1795.5 BCE p=0.13 Z
I19220 | 1794.5 BCE p=0.72 Z
I5377 | 1794 BCE p=0.71 Z
I1774 | 1753 BCE p=0.85 Z
I6680 | 1747.5 BCE p=0.36 Z
I2464 | 1645 BCE p=0.83
I7570 | 1635 BCE p=0.61 Z
I7573 | 1566 BCE p=0.67 Z
I7569 | 1534 BCE p=0.95 Z
I2639 | 1483.5 BCE p=0.15 Z
I19858 | 1481.5 BCE p=0.92 Z
dfr003 | 1479.5 BCE p=0.10 Z
I13714 | 1475 BCE p=0.15 Z
I16403 | 1475 BCE p=0.71 Z
I19857 | 1471.5 BCE p=0.67 Z
I19915 | 1470.5 BCE p=0.51 Z
I19859 | 1453.5 BCE p=0.27 Z
I19860 | 1452 BCE p=0.19 Z
I7568 | 1450 BCE p=0.73 Z
muw005 | 1420 BCE p=0.58
I3082 | 1412.5 BCE p=0.26 Z
I7572 | 1406.5 BCE p=0.41 Z
I7579 | 1399 BCE p=0.90 Z
I2458 | 1390.5 BCE p=0.49 Z
I7571 | 1374.5 BCE p=0.07 Z
dfr001 | 1373 BCE p=0.45
I7640 | 1356 BCE p=0.23 Z
I19913 | 1317 BCE p=0.23 Z
I7574 | 1317 BCE p=0.78 Z
I7627 | 1317 BCE p=0.48 Z
I13710 | 1307 BCE p=0.75 Z
I7626 | 1264.5 BCE p=0.52 Z
I13716 | 1260 BCE p=0.65 Z
I7577 | 1254.5 BCE p=0.49 Z
I2448 | 1250 BCE p=0.03 Z
I16454 | 1218.5 BCE p=0.74 Z
I7575 | 1142.5 BCE p=0.28 Z
I7580 | 1136.5 BCE p=0.15 Z
I7578 | 1134.5 BCE p=0.88 Z
I13617 | 1133 BCE p=0.58 Z
I7576 | 1107 BCE p=0.68 Z
I7628 | 1097.5 BCE p=0.12 Z
I7629 | 1067 BCE p=0.09 Z
I16469 | 995 BCE p=0.41 Z
I5383 | 995 BCE p=0.23 Z
I12936 | 995 BCE p=0.72 Z
I13711 | 984 BCE p=0.35 Z
I13712 | 963.5 BCE p=0.69 Z
I13713 | 946 BCE p=0.12 Z
I14742 | 935.5 BCE p=0.32 Z
I14377 | 925 BCE p=0.84 Z
I14864 | 899.5 BCE p=0.36 Z
I14862 | 897 BCE p=0.31 Z
I14865 | 889 BCE p=0.95 Z
I14861 | 860 BCE p=0.08 Z
I14358 | 859.5 BCE p=0.43 Z
I14379 | 855 BCE p=0.32 Z
I12624 | 850 BCE p=0.37 Z
0
20
40
60
80
100%
Admixture proportion

Supplementary Figure 16. Two-way qpAdm modelling of Bronze Age individuals as a mixture of Local Neolithic and Bell Beaker Germany sources. The plot includes Bronze Age individuals from this study together with available English Bronze Age comparative individuals passing the >30,000 SNP target threshold. Samples are ordered chronologically from oldest to youngest. Blue labels indicate English comparative individuals; black labels indicate individuals from this study. Bold labels indicate individuals with >50,000 SNPs, while regular labels indicate individuals below this threshold. Bars show estimated ancestry proportions, with Local Neolithic shown in green and Bell Beaker Germany in orange. Error bars indicate 95% confidence intervals. P-values are shown next to each sample and are coloured when the 95% confidence interval for the Local Neolithic source crosses zero. Asterisks indicate non-feasible two-way qpAdm models.

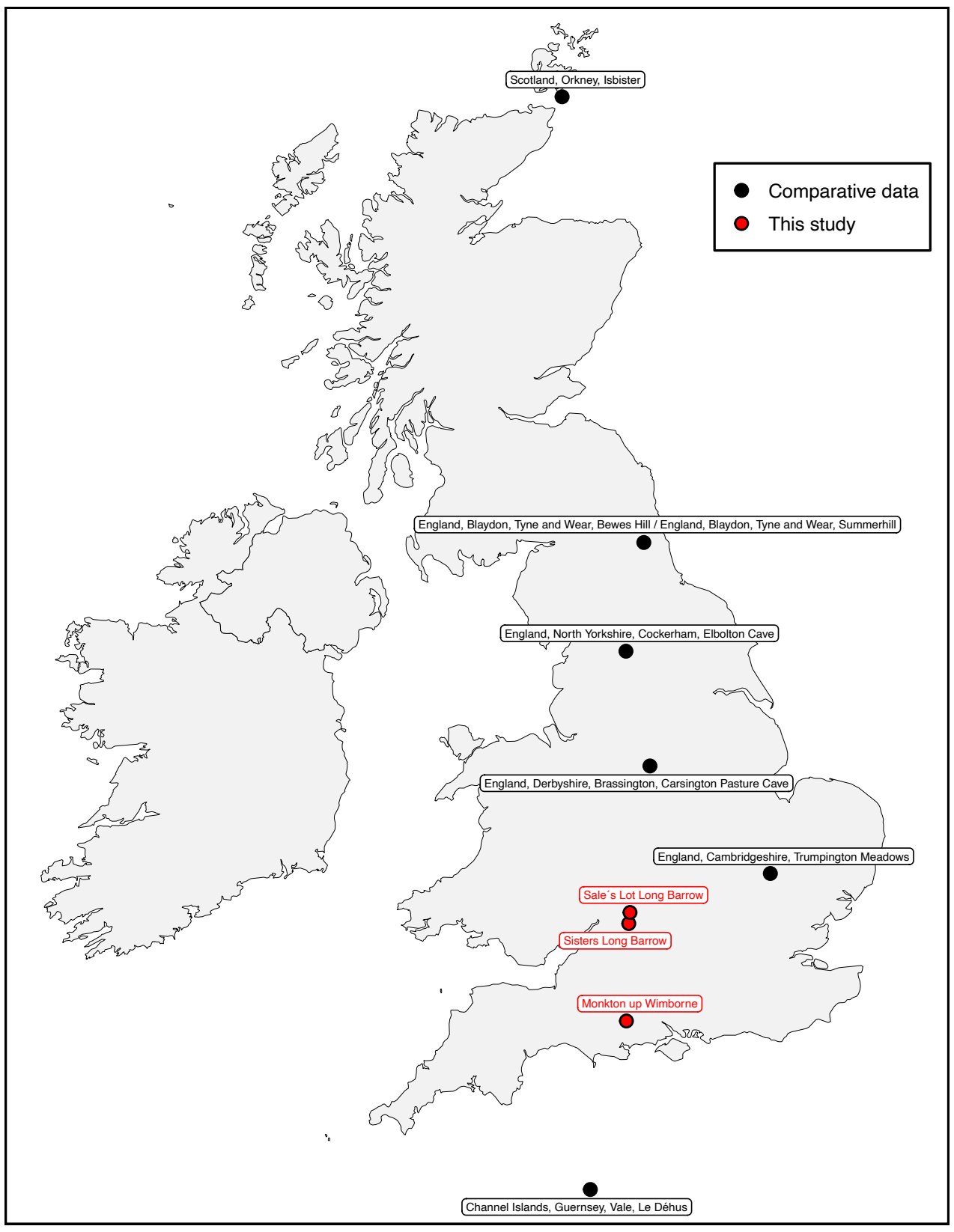

Supplementary Figure 17. Reuse of burial localities in the British and Irish Isles. Shown are sites containing individuals with direct radiocarbon dates spanning both the Neolithic (5500–2550 BCE) and the Chalcolithic/Bronze Age (2550–800 BCE). Sites from this study are highlighted in red, while comparative sites are shown in black. Reuse is inferred solely from chronological evidence not genetic affinity.

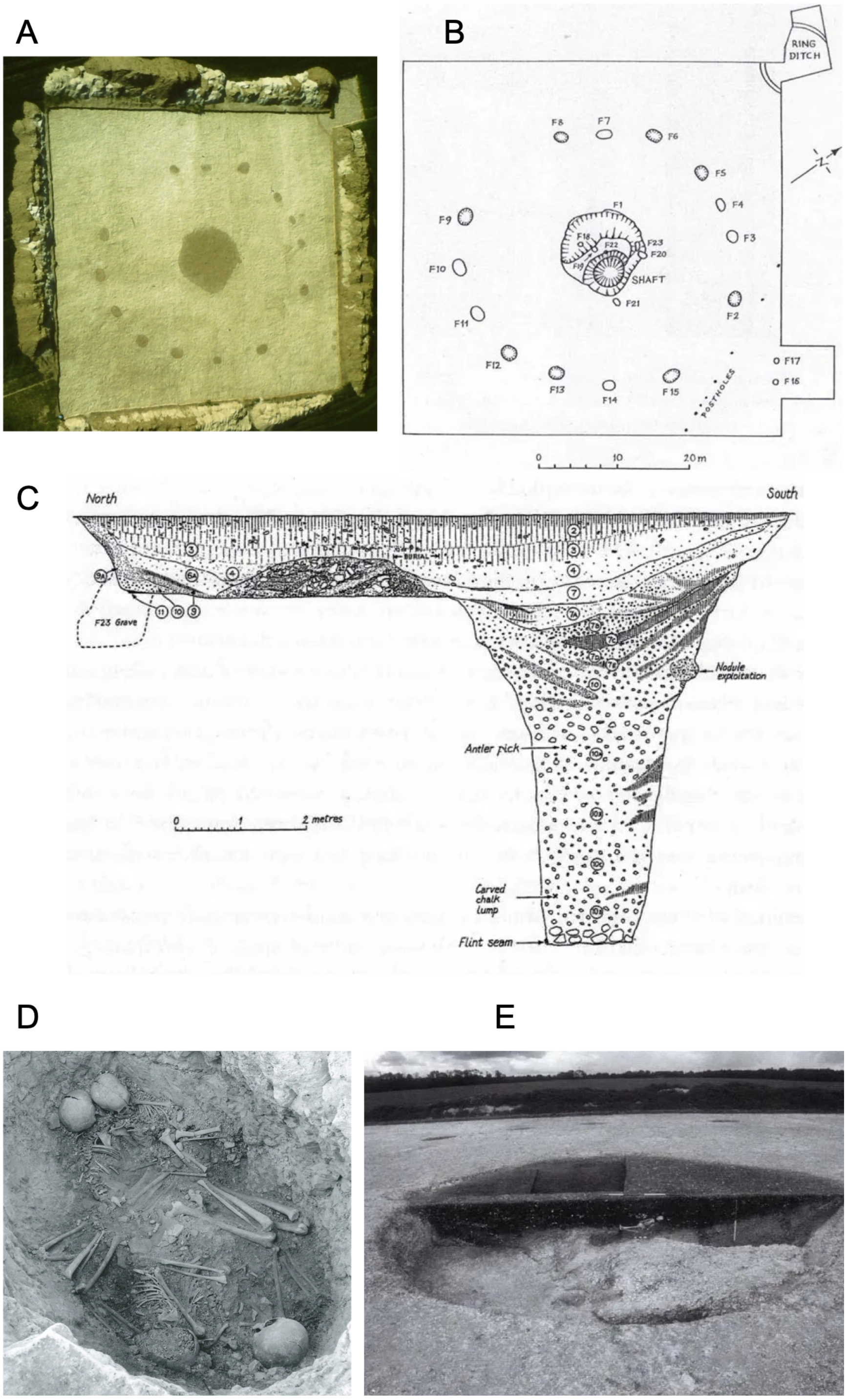

Supplementary Figure 18. Overview of the Monkton-up-Wimborne monument complex, illustrating the principal structural elements and burial phases[17]. **A**, Aerial view of the MUW97 monument (© Martin Green). **B**, Plan of the feature. **C**, Section through the central pit/shaft and associated chalk-rubble platform. **D**, Middle Neolithic co-burial of multiple individuals (photo: © Dave Webb). **E**, Early Bronze Age burial visible centrally below the horizontal range pole; the vertical range pole marks the shaft and part of the chalk-rubble platform is visible in the foreground (© Martin Green).

Supplementary references

1. Transactions of the Bristol and Gloucestershire Archaeological Society 134 (2016): 270; 135 (2017): 316; 136 (2018): 317; 137 (2019): 269; 138 (2020): 330; 139 (2021): 308; 140 (2022): 278.
2. Clark, G. Excavations on Defence Sites, 1939–1945. I. Mainly Neolithic – Bronze Age. By W. F. Grimes. H.M. Stationery Office, London, 1960. *Proc. Prehist. Soc.* **27**, 352–353 (1961).
3. Grimes, W. F. Excavations on Defence Sites, 1939–1945. Vol. I: Mainly Neolithic–Bronze Age. Ministry of Works Archaeological Reports No. 3 (HMSO, London, 1960).
4. O'Neil, H. E. Sale's Lot long barrow, Withington, Gloucestershire, 1962–1965. Transactions of the Bristol and Gloucestershire Archaeological Society 85, 5–35 (1966).
5. Darvill, T. & Grinsell, L. V. Gloucestershire barrows: supplement 1961–1988. Transactions of the Bristol and Gloucestershire Archaeological Society 107, 39–105 (1989).
6. Smith, M. & Brickley, M. The date and sequence of use of neolithic funerary monuments: New AMS dating evidence from the Cotswold-Severn region. *Oxford J Archaeology* **25**, 335–355 (2006).
7. Pitt-Rivers, A. H. L. F. Excavations in Cranborne Chase, near Rushmore, on the Borders of Dorset and Wilts, 1893–1896. Vol. IV (privately printed, 1898).
8. Allen, M. J. et al. Wor Barrow, Cranborne Chase, Dorset: Chronological Modelling. Historic England Research Report Series 9-2016 (Historic England, Portsmouth, 2016). doi:10.5284/1046423.
9. Kinnes, I. A. *Non-Megalithic Long Barrows and Allied Structures in the British Neolithic*. (Department of Prehistoric and Romano-British Antiquities, London, 1992).
10. Kinnes, I. *Round Barrows and Ring-Ditches in the British Neolithic*. (British Museum, London, 1979).
11. Barrett, J. C., Bradley, R. & Green, M. Landscape, Monuments, and Society: The Prehistory of Cranborne Chase (Cambridge University Press, Cambridge, 1991).
12. Bailey, L., Green, M. & Smith, M. J. Keeping the family together: Canada Farm's Bronze Age burials. Current Archaeology 279, 20–26 (2013).
13. Smith, M. J. *et al.* Holding on to the past: Southern British evidence for mummification and retention of the dead in the Chalcolithic and Bronze Age. *Journal of Archaeological Science: Reports* **10**, 744–756 (2016).
14. Delbarre, G., Pollard, J., Green, M., Smith, M., Endicott, P. & Allen, M. Knowle Hill Farm, Dorset, UK: a Chalcolithic (very) cold case? PAST 92, 14–15 (2019).
15. Green, M. & Allen, M. J. *A Landscape Revealed: 10,000 Years on a Chalkland Farm*. (Tempus, Stroud, 2000).

16. Richards, J. Meet the Ancestors: Unearthing the Evidence That Brings Us Face to Face with the Past (BBC Worldwide, London, 1999).
17. French, C., Lewis, H., Allen, M. J., Green, M., Scaife, R. & Gardiner, J. Prehistoric Landscape Development and Human Impact in the Upper Allen Valley, Cranborne Chase, Dorset (McDonald Institute for Archaeological Research, Cambridge, 2007).
18. Atkinson, R. J. C. The Dorset Cursus. *Antiquity* **29**, 4–9 (1955).
19. Penny, A. & Wood, J. The Dorset Cursus complex—a Neolithic astronomical observatory? Archaeological Journal 130(1), 44–76 (1973).
20. Bowden, M., Bradley, R., Gaffney, V. & Mepham, L. The date of the Dorset Cursus. Proceedings of the Prehistoric Society 49(1), 376–379 (1983). doi:10.1017/S0079497X00008069.
21. Bradley, R., Cleal, R., Gardiner, J., Legge, A., Raymond, F. & Thomas, J. Sample excavation on the Dorset Cursus, 1984. Proceedings of the Dorset Natural History and Archaeological Society 106, 128–132 (1984).
22. Yang, D. Y., Eng, B., Waye, J. S., Dudar, J. C. & Saunders, S. R. Improved DNA extraction from ancient bones using silica-based spin columns. *Am. J. Phys. Anthropol.* **105**, 539–543 (1998).
23. Damgaard, P. B. *et al.* Improving access to endogenous DNA in ancient bones and teeth. *Sci Rep* **5**, 11184 (2015).
24. Günther, T. *et al.* Population genomics of Mesolithic Scandinavia: Investigating early postglacial migration routes and high-latitude adaptation. *PLoS Biol* **16**, e2003703 (2018).
25. Malmström, H. *et al.* More on Contamination: The Use of Asymmetric Molecular Behavior to Identify Authentic Ancient Human DNA. *Molecular Biology and Evolution* **24**, 998–1004 (2007).
26. Günther, T. *et al.* Ancient genomes link early farmers from Atapuerca in Spain to modern-day Basques. *Proc. Natl. Acad. Sci. U.S.A.* **112**, 11917–11922 (2015).
27. Meyer, M. & Kircher, M. Illumina Sequencing Library Preparation for Highly Multiplexed Target Capture and Sequencing. *Cold Spring Harb Protoc* **2010**, pdb.prot5448 (2010).
28. Lindgreen, S. AdapterRemoval: easy cleaning of next-generation sequencing reads. *BMC Res Notes* **5**, 337 (2012).
29. Schubert, M., Lindgreen, S. & Orlando, L. AdapterRemoval v2: rapid adapter trimming, identification, and read merging. *BMC Res Notes* **9**, 88 (2016).
30. Martin, M. Cutadapt removes adapter sequences from high-throughput sequencing reads. *EMBnet j.* **17**, 10 (2011).
31. Magoč, T. & Salzberg, S. L. FLASH: fast length adjustment of short reads to improve genome assemblies. *Bioinformatics* **27**, 2957–2963 (2011).

32. Li, H. & Durbin, R. Fast and accurate short read alignment with Burrows–Wheeler transform. *Bioinformatics* **25**, 1754–1760 (2009).
33. Danecek, P. *et al.* Twelve years of SAMtools and BCFtools. *GigaScience* **10**, giab008 (2021).
34. Fu, Q. *et al.* A Revised Timescale for Human Evolution Based on Ancient Mitochondrial Genomes. *Current Biology* **23**, 553–559 (2013).
35. Green, R. E. *et al.* A Draft Sequence of the Neandertal Genome. *Science* **328**, 710–722 (2010).
36. Skoglund, P., Storå, J., Götherström, A. & Jakobsson, M. Accurate sex identification of ancient human remains using DNA shotgun sequencing. *Journal of Archaeological Science* **40**, 4477–4482 (2013).
37. Weissensteiner, H. *et al.* HaploGrep 2: mitochondrial haplogroup classification in the era of high-throughput sequencing. *Nucleic Acids Res* **44**, W58–W63 (2016).
38. Van Oven, M. PhyloTree Build 17: Growing the human mitochondrial DNA tree. *Forensic Science International: Genetics Supplement Series* **5**, e392–e394 (2015).
39. Brunel, S. *et al.* Ancient genomes from present-day France unveil 7,000 years of its demographic history. *Proc. Natl. Acad. Sci. U.S.A.* **117**, 12791–12798 (2020).
40. Gamba, C. *et al.* Genome flux and stasis in a five millennium transect of European prehistory. *Nat Commun* **5**, 5257 (2014).
41. Olalde, I. *et al.* The Beaker phenomenon and the genomic transformation of northwest Europe. *Nature* **555**, 190–196 (2018).
42. Fernandes, D. M. *et al.* The spread of steppe and Iranian-related ancestry in the islands of the western Mediterranean. *Nat Ecol Evol* **4**, 334–345 (2020).
43. Mittnik, A. *et al.* Kinship-based social inequality in Bronze Age Europe. *Science* **366**, 731–734 (2019).
44. Olalde, I. *et al.* The genomic history of the Iberian Peninsula over the past 8000 years. *Science* **363**, 1230–1234 (2019).
45. Allentoft, M. E. *et al.* Population genomics of Bronze Age Eurasia. *Nature* **522**, 167–172 (2015).
46. Cassidy, L. M. *et al.* A dynastic elite in monumental Neolithic society. *Nature* **582**, 384–388 (2020).
47. Sánchez-Quinto, F. *et al.* Megalithic tombs in western and northern Neolithic Europe were linked to a kindred society. *Proc. Natl. Acad. Sci. U.S.A.* **116**, 9469–9474 (2019).
48. Mathieson, I. *et al.* Genome-wide patterns of selection in 230 ancient Eurasians. *Nature* **528**, 499–503 (2015).
49. Narasimhan, V. M. *et al.* The formation of human populations in South and Central Asia. *Science* **365**, eaat7487 (2019).

50. Mathieson, I. *et al.* The genomic history of southeastern Europe. *Nature* **555**, 197–203 (2018).
51. Brace, S. *et al.* Ancient genomes indicate population replacement in Early Neolithic Britain. *Nat Ecol Evol* **3**, 765–771 (2019).
52. Fu, Q. *et al.* The genetic history of Ice Age Europe. *Nature* **534**, 200–205 (2016).
53. Rivollat, M. *et al.* Ancient genome-wide DNA from France highlights the complexity of interactions between Mesolithic hunter-gatherers and Neolithic farmers. *Sci. Adv.* **6**, eaaz5344 (2020).
54. Lazaridis, I. *et al.* Ancient human genomes suggest three ancestral populations for present-day Europeans. *Nature* **513**, 409–413 (2014).
55. Antonio, M. L. *et al.* Ancient Rome: A genetic crossroads of Europe and the Mediterranean. *Science* **366**, 708–714 (2019).
56. Jones, E. R. *et al.* Upper Palaeolithic genomes reveal deep roots of modern Eurasians. *Nat Commun* **6**, 8912 (2015).
57. Patterson, N. *et al.* Large-scale migration into Britain during the Middle to Late Bronze Age. *Nature* **601**, 588–594 (2022).
58. Mallick, S. et al. The Allen Ancient DNA Resource (AADR) a curated compendium of ancient human genomes. Sci Data 11, 182 (2024).
59. The 1000 Genomes Project Consortium. A global reference for human genetic variation. *Nature* **526**, 68–74 (2015).
60. Mallick, S. *et al.* The Simons Genome Diversity Project: 300 genomes from 142 diverse populations. *Nature* **538**, 201–206 (2016).
61. Bergström, A. *et al.* Insights into human genetic variation and population history from 929 diverse genomes. *Science* **367**, eaay5012 (2020).
62. Purcell, S. *et al.* PLINK: A Tool Set for Whole-Genome Association and Population-Based Linkage Analyses. *The American Journal of Human Genetics* **81**, 559–575 (2007).
63. Patterson, N., Price, A. L. & Reich, D. Population Structure and Eigenanalysis. *PLoS Genet* **2**, e190 (2006).
64. Price, A. L. *et al.* Principal components analysis corrects for stratification in genome-wide association studies. *Nat Genet* **38**, 904–909 (2006).
65. RStudio Team. RStudio: Integrated Development for R. RStudio, PBC, Boston, MA URL http://www.rstudio.com/. (2020).
66. R Core Team. R: A Language and Environment for Statistical Computing. R Foundation for Statistical Computing, Vienna, Austria. https://www.R-project.org/. (2023).
67. Alexander, D. H., Novembre, J. & Lange, K. Fast model-based estimation of ancestry in unrelated individuals. *Genome Res.* **19**, 1655–1664 (2009).

68. Behr, A. A., Liu, K. Z., Liu-Fang, G., Nakka, P. & Ramachandran, S. pong: fast analysis and visualization of latent clusters in population genetic data. *Bioinformatics* **32**, 2817–2823 (2016).
69. Alaçamlı, E. *et al.* READv2: advanced and user-friendly detection of biological relatedness in archaeogenomics. *Genome Biol* **25**, 216 (2024).
70. Monroy Kuhn, J. M., Jakobsson, M. & Günther, T. Estimating genetic kin relationships in prehistoric populations. *PLoS ONE* **13**, e0195491 (2018).
71. Skoglund, P. *et al.* Genetic evidence for two founding populations of the Americas. *Nature* **525**, 104–108 (2015).
72. Kolde, R. pheatmap: Pretty Heatmaps. R package version 1.0.12 (2019).
73. Scheib, C. L. *et al.* East Anglian early Neolithic monument burial linked to contemporary Megaliths. *Annals of Human Biology* **46**, 145–149 (2019).
74. Cassidy, L. M. *et al.* Neolithic and Bronze Age migration to Ireland and establishment of the insular Atlantic genome. *Proc. Natl. Acad. Sci. U.S.A.* **113**, 368–373 (2016).
75. Fowler, C. *et al.* A high-resolution picture of kinship practices in an Early Neolithic tomb. *Nature* **601**, 584–587 (2022).
76. Maier, R. *et al.* On the limits of fitting complex models of population history to f-statistics. *eLife* **12**, e85492 (2023).
77. Patterson, N. *et al.* Ancient Admixture in Human History. *Genetics* **192**, 1065–1093 (2012).
78. Haak, W. *et al.* Massive migration from the steppe was a source for Indo-European languages in Europe. *Nature* **522**, 207–211 (2015).
79. Harney, É., Patterson, N., Reich, D. & Wakeley, J. Assessing the performance of qpAdm: a statistical tool for studying population admixture. *Genetics* **217**, iyaa045 (2021).
80. Li, J. Z. *et al.* Worldwide Human Relationships Inferred from Genome-Wide Patterns of Variation. *Science* **319**, 1100–1104 (2008).
81. Jakobsson, M. *et al.* Genotype, haplotype and copy-number variation in worldwide human populations. *Nature* **451**, 998–1003 (2008).
82. Llorente, M. G. *et al.* Ancient Ethiopian genome reveals extensive Eurasian admixture in Eastern Africa. *Science* **350**, 820–822 (2015).
83. Raghavan, M. *et al.* Upper Palaeolithic Siberian genome reveals dual ancestry of Native Americans. *Nature* **505**, 87–91 (2014).
84. Lazaridis, I. *et al.* The genetic history of the Southern Arc: A bridge between West Asia and Europe. *Science* **377**, eabm4247 (2022).
85. Needham, S. Chronology and periodisation in the British Bronze Age. *Acta Archaeologica* **67**, 121–140 (1996).
86. Barber, M. *Bronze and the Bronze Age : Metalwork and Society in Britain c.2500-800 BC*. (Stroud : Tempus Publishing, 2003).

87. Bronk Ramsey, C. Bayesian Analysis of Radiocarbon Dates. *Radiocarbon* **51**, 337–360 (2009).
88. Reimer, P. J. *et al.* The IntCal20 Northern Hemisphere Radiocarbon Age Calibration Curve (0–55 cal kBP). *Radiocarbon* **62**, 725–757 (2020).
89. Martin, J. C. GeoJSON & TopoJSON collection of UK boundary data. Generalised clipped boundaries provided by the Office for National Statistics, Ordnance Survey, and Ordnance Survey of Northern Ireland. Converted to GeoJSON using ogr2ogr, and TopoJSON using topojson. http://martinjc.github.io/UK-GeoJSON/ (2016).
